# Genomic and structural insights into the high-efficiency poly(3-hydroxybutyrate) biodegradation in *Terrabacter* sp. AAH1

**DOI:** 10.3389/fmicb.2026.1891347

**Published:** 2026-07-30

**Authors:** Sunho Park, Ji Hyuk Ko, Chaeyeon Yang, Seohyun Jeong, Jisu Kim, Seunghui Kwak, Hyunji Lee, Subin Yook, Chunghwan Baek, Jae Young Lee, Taegun Seo

**Affiliations:** Department of Life Science, Dongguk University–Seoul, Goyang, Republic of Korea

**Keywords:** biodegradation, functional genomics, molecular docking, PHB depolymerase, poly(3-hydroxybutyrate), *Terrabacter* sp. AAH1

## Abstract

The freshwater isolate *Terrabacter* sp. AAH1 exhibits highly efficient poly(3-hydroxybutyrate) (PHB) biodegradation, achieving 98.02 ± 0.08% weight loss within 12 days. Scanning electron microscopy and Fourier-transform infrared spectroscopy demonstrated that this rapid disintegration is driven by extensive surface erosion and the hydrolytic cleavage of ester bonds. Comparative genomic analysis across related taxa revealed that while putative PHB depolymerase genes are present in a subset of *Terrabacter* and allied genera, strain AAH1 is distinguished by its candidate degradation phenotype. The strain’s genomic architecture is predicted to integrate a secreted putative PHB depolymerase with a predict metabolic suite for the degradation of PHB via 3-HB oxidation, SCOT-mediated acetoacetate activation, and β-oxidation-like pathway converging on the TCA cycle. *In silico* structural modeling and molecular docking further supported a hypothetical compartmentalized degradation system, in which the Sec-type secreted putative depolymerase *Te*_EPD is proposed to initiate extracellular PHB hydrolysis, while *Te*_YbfF, *Te*_AES1, and *Te*_AES2 predicted to lack signal peptides are tentatively assigned putative intracellular roles. Among the four candidates, *Te*_EPD exhibited a predicted binding affinity of –4.9 kcal/mol for the PHB trimer via a conserved Ser–Asp–His (S–D–H) catalytic triad, and was uniquely classified within the extracellular short-chain-length PHA depolymerase type 1 (e_dPHAscl_type1) family based on domain annotation and sequence motif analysis. By proposing a putative association between specific genomic features and macroscopic polymer degradation, this study suggests that *Terrabacter* sp. AAH1 may represent a candidate biocatalyst warranting further investigation for potential applications in bioplastic waste management.

## 1 Introduction

Poly(3-hydroxybutyrate) (PHB), a representative member of the polyhydroxyalkanoate (PHA) family, has attracted considerable attention as a biodegradable alternative to petroleum-based plastics (Verlinden et al., 2007; Geyer et al., 2017; Sirohi et al., 2020). PHB exhibits thermal and mechanical properties comparable to those of conventional plastics while being completely biodegradable under appropriate environmental conditions (Verlinden et al., 2007; Cho et al., 2021; Julinová et al., 2023). Since the foundational studies in the late 20th century, it has been established that the environmental fate of PHB depends heavily on its crystalline structure and the presence of specific microbial enzymes (Jendrossek et al., 1996; Iwata et al., 1997; Hiraishi et al., 2010). However, despite its intrinsic biodegradability, PHB often demonstrates limited or inconsistent degradation rates in natural environments (Julinová et al., 2023; Wang et al., 2024; Chhetri et al., 2025). This limitation is primarily attributed to its high crystallinity and hydrophobic surface characteristics, which restrict enzyme accessibility and reduce depolymerization efficiency (Hiraishi et al., 2010; Park et al., 2021). Therefore, understanding the degradation behavior of PHB films at the material level and identifying efficient microbial systems capable of penetrating and hydrolyzing the polymer matrix are essential for improving its environmental performance.

Microbial degradation of PHB is predominantly mediated by extracellular PHB depolymerases, which hydrolyze the insoluble polymer into soluble oligomers or 3-hydroxybutyrate (3-HB) monomers via a conserved S–D–H catalytic triad within an α/β-hydrolase fold (Jendrossek et al., 1996; Iwata et al., 1997; Hiraishi et al., 2010). To date, PHB biodegradation has been extensively studied in genera such as *Bacillus*, *Pseudomonas*, *Streptomyces*, and *Ralstonia* (Iwata et al., 1997; Cho et al., 2021; Chhetri et al., 2025). In contrast, members of the phylum Actinobacteria, despite their ecological importance as major soil decomposers, have received comparatively less attention in the context of PHB depolymerization. Although some actinomycetes like *Streptomyces* have been identified as PHA degraders (Martínez et al., 2015; Chhetri et al., 2025), the molecular and genomic basis of PHB turnover in the genus *Terrabacter* remains poorly understood. The genus *Terrabacter*, belonging to the family *Intrasporangiaceae*, comprises Gram-positive actinomycetes characterized by high G+C contents and substantial metabolic versatility (Collins et al., 1989; Weon et al., 2007; Lee et al., 2008). Previous studies indicate that *Terrabacter* species are capable of degrading recalcitrant organic substrates (Takagi et al., 2002; Tappe et al., 2015), suggesting a specialized enzymatic repertoire that may include candidate hydrolases for synthetic and natural polymers.

Recent advances in whole-genome sequencing and protein structure prediction tools have enabled integrative approaches for elucidating biodegradation mechanisms. Structural modeling and molecular docking simulations allow prediction of enzyme–substrate interactions even when experimentally determined protein structures are unavailable. When combined with morphological analyses such as scanning electron microscopy (SEM) and chemical characterization using Fourier-transform infrared (FT-IR) spectroscopy, these approaches provide a comprehensive framework for correlating macroscopic material degradation with molecular-level enzymatic processes (Julinová et al., 2023; Jašek et al., 2025).

In this study, we investigated the biodegradation behavior and underlying genomic potential of *Terrabacter* sp. AAH1, a high-efficiency strain isolated from a freshwater environment. Recognizing the limitations of relying solely on predictive data, we employed an integrative approach: the degradation phenotype was quantitatively assessed through gravimetric weight loss and cross-verified via SEM and FT-IR (Chang et al., 2024). Whole-genome sequencing was utilized for the genomic mining of candidate esterases and to map the predicted metabolic pathways for PHB degradation (Collins et al., 1989). Comparative genomic analysis was performed to assess the distribution of candidate depolymerase genes among related species (Kuroda et al., 2025).

Furthermore, *in silico* structural modeling and molecular docking simulations were employed to explore the potential interactions between the catalytic triad of putative depolymerases and PHB oligomer models. By correlating macroscopic material characterization with genomic insights, this study aims to elucidate the mechanistic basis of efficient PHB depolymerization in *Terrabacter* sp. AAH1 and provides new perspectives on the role of actinobacteria in the global polymer cycle.

## 2 Materials and methods

### 2.1 Materials

All chemicals used in this study were of analytical grade. The PHB granules were purchased from Goodfellow Cambridge Ltd. (Huntingdon, United Kingdom). Chloroform and dichloromethane (DCM), used for preparing the PHB emulsions and films, were obtained from Daejung Chemicals & Metals Co., Ltd. (Siheung, Republic of Korea), while Sarkosyl NL was purchased from Sigma-Aldrich^®^ (Merck KGaA, Darmstadt, Germany). Glucose, glycerol, lactose, fructose, and sucrose used as carbon sources were purchased from BioShop Canada Inc. (Ontario, Canada). Casein, chitin, Tween 20, and Tween 80 used in hydrolysis assays were obtained from Biopure (Namyangju, Republic of Korea).

### 2.2 Preparation of amorphous poly(3-hydroxybutyrate) emulsions and crystalline films

The method previously described by Shin et al. (2024) was adapted with minor modifications to prepare the PHB emulsions and films. For emulsion preparation, 4 g of PHB granules were dissolved in 80 mL of DCM in a water bath maintained at 40°C for at least 4 h, until the granules were no longer visible. Subsequently, the PHB–DCM solution and 4 mL of 2% (w/v) Sarkosyl NL surfactant were added to 200 mL of distilled water. The mixture was sonicated for 10 min using a pulsed cycle of 15 s on and 45 s off at 85% amplitude. Subsequently, the emulsion was stirred for 24 h using a magnetic stirrer at 40°C inside a fume hood to facilitate complete removal of residual DCM, which could potentially inhibit cell growth. After solvent evaporation, 400 mL of distilled water was added and mixed thoroughly to obtain a 1% (w/v) amorphous PHB emulsion, which was stored at 4°C in the dark until use for screening assays.

To prepare PHB-containing agar plates, Reasoner’s 2A (R2A) medium (MB Cell, Smart Science Co., Ltd., Bangkok, Thailand) supplemented with casamino acid hydrolysate (0.5 g/L), yeast extract (0.5 g/L), proteose peptone (0.5 g/L), dextrose (0.5 g/L), soluble starch (0.5 g/L), potassium phosphate (0.3 g/L), magnesium sulfate (0.03 g/L), sodium pyruvate (0.3 g/L), and agar (15 g/L) was mixed with PHB emulsion at a final concentration of 100 mL/L. The use of R2A as a basal medium was intended to provide minimal nutrients to support initial bacterial establishment while ensuring that the observable clear zones were a result of polymer depolymerization. The resulting R2A agar containing 0.1% (w/v) PHB was autoclaved and used for bacterial cultivation.

The PHB films were prepared using the solvent-casting method described by Chhetri et al. (2026). Briefly, 0.5 g of PHB granules were completely dissolved in 50 mL of chloroform at 55°C for 9 h. The resulting solution was poured into a glass petri dish and dried in a fume hood until solvent evaporation was complete, yielding a uniform crystalline PHB film. To prevent unstable PHB film formation caused by strong airflow, the glass petri dish was covered with a beaker during drying. Prior to inoculation, the PHB films were immersed in the appropriate broth and sterilized by autoclaving at 121°C for 15 min. The structural integrity of the sterilized films was visually confirmed before use in degradation experiments.

### 2.3 Isolation and identification of the PHB-degrading bacterium

Freshwater samples collected from an agricultural stream in Paju, Republic of Korea (37°41’54.68” N, 126°48’13.25” E) were transported on ice and suspended in sterile 0.85% (w/v) saline. The suspensions were serially diluted (10^–1^–10^–2^) and spread onto R2A agar plates supplemented with 0.1% (w/v) PHB emulsion (Park et al., 2025). After incubation at 30°C for 7 days, colonies exhibiting distinct clear halos were isolated and preserved at –80°C in a 50% (v/v) glycerol suspension.

The 16S rRNA gene was amplified and sequenced using universal primers (27F and 1492R) by Sanger sequencing (SolGent Co., Ltd., Daejeon, Republic of Korea), yielding a sequence of 1,390 bp covering nearly the full length of the gene. The assembled sequence was identified using the EzBioCloud database (Yoon et al., 2017) and deposited in the National Center for Biotechnology Information (NCBI) database under accession number PV746212 (National Library of Medicine, Bethesda, MD, United States).^1^

For phylogenetic analysis, reference 16S rRNA gene sequences of closely related taxa were retrieved from the EzBioCloud database. All reference sequences included in the analysis were near-full-length, ranging from approximately 1,400 to 1,440 bp, ensuring comparability with the sequence of strain AAH1 (1,390bp). Multiple sequence alignment was performed using ClustalW in MEGA X (Kumar et al., 2018). A maximum-likelihood tree was constructed based on the Kimura 2-parameter model with 1,000 bootstrap replicates to assess phylogenetic relationships and ensure statistical reliability (Kimura, 1980; Guindon and Gascuel, 2003).

The extracellular enzymatic activity of strain AAH1 was evaluated by assessing its hydrolytic potential against various polymeric substrates. R2A agar plates were supplemented with 1% (w/v) starch, 2.8% (w/v) skim milk, 1% (w/v) carboxymethyl cellulose, 1% (w/v) chitin, and 1% (v/v) Tween 20/80 to assay for amylase, protease, cellulase, chitinase, and lipase activities, respectively (Park et al., 2024). After incubation at 30°C for 4 days, the resulting hydrolysis zones were visualized directly or by staining with 0.1 N iodine for amylase and Congo red for cellulase (Sazci et al., 1986; Yassin et al., 2021; Afrin et al., 2024). Additionally, cell morphology was examined using a transmission electron microscope (JEM-1010, JEOL Ltd., Tokyo, Japan) following negative staining with 3% uranyl acetate to characterize the physiological features of the isolate.

### 2.4 Optimization of PHB biodegradation and gravimetric analysis

The optimal growth medium for PHB-degrading enzyme production was determined by culturing strain AAH1 on marine agar (MA; MB Cell, Smart Science Co., Ltd., Seoul, Republic of Korea), R2A agar, nutrient agar (NA; Difco™, Becton, Dickinson and Company, Franklin Lakes, NJ, United States), tryptic soy agar (TSA; Difco™, Dickinson and Company, Franklin Lakes, NJ, United States), and Luria-Bertani (LB) agar (Difco™, Dickinson and Company, Franklin Lakes, NJ, United States), each supplemented with 0.1% (w/v) PHB emulsion. The radius of the clear zone was measured after incubation at 30°C for 7 days to evaluate PHB-degrading activity. R2A was selected as the optimal basal medium for subsequent experiments, as its oligotrophic nature facilitated superior clear zone resolution to nutrient-rich alternatives. Additionally, the assay was performed using the medium selected for optimal growth, testing the strain under various conditions: temperatures (15°C, 20°C, 25°C, 30°C, and 35°C), NaCl concentrations (0–3.0% w/v, in 1.0% increments), and pH values (5.0–12.0, in 1.0-unit increments).

The effect of additional carbon sources on the PHB-degrading efficiency of strain AAH1 was evaluated using the optimal medium sup plemented with 1% (w/v) fructose, glucose, glycerol, sucrose, or lactose. A 6.0 mm paper disk (Advantec^®^, Toyo Roshi Kaisha, Ltd., Tokyo, Japan) was placed at the center of the PHB-containing optimal medium. Strain AAH1 was pre-cultured in R2A broth at 30°C for 3 days. Subsequently, 10 μL of the pre-cultured broth was inoculated onto the paper disk, and the plates were incubated at 30°C for 12 days. The formation of clear zones was monitored at 3-day intervals by measuring the radius from the center of the 6 mm paper disk. All experiments were performed in triplicate.

For the liquid-phase degradation assay, a 20 mg piece of the prepared PHB film was placed in 50 mL of R2A broth in a 125 mL flask, and autoclaved at 121°C for 15 min. The film’s structural integrity post-sterilization was confirmed visually. Subsequently, 1 mL of the 3-day-old pre-culture was inoculated into the broth and incubated in a rotary shaker at 30°C and 180 rpm for 12 days. Culture samples were collected at 2, 4, 6, 8, and 12-day intervals to evaluate PHB film biodegradation. The uninoculated control, consisting of R2A broth with a PHB film, was incubated under identical conditions.

At each sampling time point, 50 mL of the culture broth was transferred to a conical tube and directly lyophilized to prevent the loss of fine PHB debris or microplastics in the supernatant. The lyophilized matrix of biomass and remaining polymer was transferred to a glass vial. Residual PHB was then extracted by adding 10 mL of chloroform and incubating the mixture in a water bath at 55°C to ensure complete dissolution. The solution was then filtered into a pre-weighed aluminum dish using a glass syringe equipped with a 0.22 μm PTFE filter. The syringe was subsequently rinsed with an additional 3 mL of chloroform to ensure complete recovery of any remaining polymer residues. The chloroform was allowed to evaporate completely overnight in a fume hood, and the weight of the re-cast PHB film was determined gravimetrically. The biodegradation efficiency was determined based on the percentage of weight loss using the following equation (1). where *W*_0_ is the initial dry weight of the PHB film, and *W*_*t*_ is the residual weight after incubation time *t*.

### 2.5 Characterization of degraded PHB films

For morphological and chemical characterization of degraded PHB films, a separate set of biodegradation experiments was conducted independently from the gravimetric assays, using identical culture conditions (R2A broth, 30°C, 180 rpm). At each sampling time point (2, 4, 6, and 8 days), the culture broth was filtered through a 150 mm filter paper (Toyo Roshi Kaisha, Ltd., Tokyo, Japan) by gravity filtration, and the residual PHB film fragments were carefully collected using forceps and transferred to 1.5 mL microcentrifuge tubes. The recovered films were stored at –80°C until further processing for SEM and FT-IR analyses as described below. Surface morphological changes in PHB films following biodegradation by strain AAH1 were investigated using A SEM (Zeiss Sigma, Carl Zeiss AG, Jena, Germany) (Smith et al., 2020). For morphological characterization, PHB film fragments recovered by gravity filtration and collected using forceps were transferred to 1.5 mL microcentrifuge tubes as described above. The recovered film fragments were gently washed twice with phosphate-buffered saline (PBS; pH 7.4) to remove non-adherent cells and medium components. Excess PBS was removed by brief centrifugation (300 × g, 30 s) to avoid mechanical disruption of the film fragments. The washed samples were then fixed at 4°C overnight in 1 mL of 2.5% (v/v) glutaraldehyde in phosphate buffer (pH 7.4). The fixed samples were dehydrated through a graded ethanol series comprising 30, 50, 60, 70, 80, 90, and 100% (v/v) absolute ethanol (Sigma-Aldrich^®^, Merck KGaA, Darmstadt, Germany). Subsequently, the samples were dried using hexamethyldisilazane within a fume hood and sputter-coated with gold at 30 mA for 140 s. Backscattered electron images were captured using SEM at an accelerating voltage of 5 kV.

To ensure the removal of adhered biomass before chemical analysis, the PHB film recovered from the culture broth was rinsed twice with distilled water, followed by two brief washes with 100% (v/v) absolute ethanol (Supelco^®^, Merck KGaA, Darmstadt, Germany). The film was air-dried at 30°C on a clean bench for 6 h and then analyzed using FT-IR spectroscopy (IdentifyIR^®^, Smiths Detection Group Ltd., Hemel Hamstead, United Kingdom) to characterize the functional groups and chemical transitions (Uda et al., 2025). FT-IR spectra were collected from four films sampled at 0, 2, 4, and 6 days. PHB films collected on days 8 and 12 could not be recovered in sufficient amounts for FT-IR analysis owing to extensive enzymatic fragmentation and the loss of material integrity resulting from high biodegradation efficiency. The spectra were recorded over the wavenumber range 4,000–650 cm^–1^ at a spectral resolution of 4 cm^–1^. The resulting spectral data were visualized as absorbance plots using R software (v 4.4.2; R Foundation for Statistical Computing, Vienna, Austria)^2^ with the *ggplot2* package^3^ library.

### 2.6 Genome sequencing, annotation, and comparative analysis

Genomic DNA from strain AAH1 was extracted by Sanigen Inc. (Seoul, Republic of Korea). Sequencing libraries were prepared from the extracted DNA and sequenced on an Illumina NextSeq platform in 2 × 300 bp paired-end mode. Raw sequencing reads were quality-filtered using Trimmomatic v0.39 (Bolger et al., 2014) to remove adapter sequences and low-quality bases. The filtered reads were assembled *de novo* using SPAdes v3.15.3 (Bankevich et al., 2012). Genome completeness and contamination were assessed using CheckM2 (v1.1.0; Australian National University, Canberra, Australia) (Chklovski et al., 2023). The assembled genome was annotated using Prokka v1.14.6 (Seemann, 2014) and further processed through the NCBI Prokaryotic Genome Annotation Pipeline (PGAP; v6.5, National Center for Biotechnology Information, Bethesda, MD, United States)^4^ (Tatusova et al., 2016). Candidate genes associated with metabolic pathways and PHB biodegradation in strain AAH1 were identified using Cluster of Orthologous Groups (COG) analysis based on the eggNOG v5.0 database (Huerta-Cepas et al., 2019). To ensure stringent functional annotation, only genes exhibiting an E-value ≤ 1 × 10^–50^ were selected for metabolic reconstruction. To investigate the evolutionary distribution of these candidate genes across related taxa, a comparative genomic analysis was performed. A total of 37 genome sequences were retrieved from the NCBI Assembly database, comprising 25 *Terrabacter* species, 5 *Pedococcus*, 5 *Intrasporangium*, 1 *Terracoccus*, and 1 *Humibacillus* strain. All retrieved genomes were annotated using Prokka v1.14.6, and functional annotation was subsequently performed using eggNOG-mapper v2.1.13 (Cantalapiedra et al., 2021) with default settings, employing DIAMOND-based sequence similarity searches against the eggNOG v5.0 database. Target genes were identified by screening the annotated output for PHB degradation-related terms in the product and description fields. The putative PHB depolymerase was additionally confirmed by the presence of the Pfam domain Esterase_phd, which is characteristic of experimentally characterized PHB depolymerases. The genomic prevalence, gene copy number, and sequence homology of target genes, expressed as bit score, were visualized as a bubble heatmap using the ggplot2 package in R (v4.4.2). It should be noted that this annotation-based approach is inherently limited by sequence divergence and genome completeness, and the absence of detectable homologs in certain genomes does not necessarily indicate the true absence of the corresponding gene function. To provide a candidate *in silico* alternative to direct biochemical characterization, putative PHB depolymerases were prioritized by systematically screening COG functional categories for esterase-related proteins. The extracellular secretion potential of these predicted esterases was evaluated by analyzing the presence of signal peptides using SignalP v6.0 (Teufel et al., 2022). The draft genome sequence of strain AAH1 has been deposited in the NCBI database under the accession number JBTJJV010000000. The 16S rRNA gene sequences extracted from the whole-genome assembly were compared to those obtained through Sanger sequencing using the ContEst16S tool (National Institute of Genetics, Mishima, Japan) (Lee et al., 2017).

To establish precise taxonomic placement, a phylogenomic tree was reconstructed using 81 single-copy bacterial core genes with the UBCG2 pipeline (Universal Bacterial Core Genes 2; Seoul National University, Seoul, Republic of Korea)^5^ (Kim et al., 2021). Draft genome sequences of reference strains used for comparison with strain AAH1 were retrieved from the NCBI Assembly database (National Center for Biotechnology Information, Bethesda, MD, United States).^6^ Genomic relatedness between strain AAH1 and validly published species of the genus *Terrabacter* was quantitatively evaluated by calculating Average Nucleotide Identity (ANI) and digital DNA–DNA hybridization (dDDH) values using FastANI v1.33 (Jain et al., 2018) and the Genome-to-Genome Distance Calculator (GGDC v3.0)^7^ (Goris et al., 2007), respectively. A circular genome map illustrating the complete genome sequence, COG functional categories, and genes associated with PHB biodegradation and metabolic pathways was generated using Circos v0.69.10 (Krzywinski et al., 2009).

### 2.7 Computational modeling and automated molecular docking

Homologous proteins of the four esterase candidates were identified using BLASTp, while multiple sequence alignment was performed with Clustal Omega to identify conserved regions (Altschul et al., 1990; Sievers et al., 2011).

Three-dimensional structures were generated using ColabFold (v.1.5.5) and used as receptors for molecular docking analyses (Mirdita et al., 2022). The predicted models were evaluated using predicted Local Distance Difference Test (pLDDT) scores, and high-confidence regions (pLDDT > 70) were considered reliable for subsequent molecular docking studies. Structural alignment was performed using PyMOL (The PyMOL Molecular Graphics System, v3.1, Schrödinger, LLC, New York, NY, United States) to examine active-site overlap (Krissinel and Henrick, 2007).

The generated protein structures were prepared for individual molecular docking experiments using AutoDockTools (ADT, v4.2, The Scripps Research Institute, La Jolla, CA, United States) (Morris et al., 2009). Preparation involved adding polar hydrogens and assigning side-chain flexibility to binding-site residues, while main-chain atoms forming α-helices were kept rigid. The structures, initially generated in PDB format, were converted to PDBQT format for molecular docking. The PHB trimer, used as an esterase substrate, was converted from SMILES to PDB format using RDKit (open-source cheminformatics toolkit).^8^ Ligand conformational constraints and partial charges were assigned using ADT (v4.2, The Scripps Research Institute, La Jolla, CA, United States), while the ligand was converted to PDBQT format.

Putative substrate-binding pockets were identified using Fpocket, and the pocket located nearest to the predicted catalytic residues was selected as the docking site. Docking grids were individually defined for each protein to encompass the predicted binding pocket together with the catalytic residues involved in substrate hydrolysis. Because the size and location of the predicted pockets varied among proteins, grid dimensions were optimized separately for each receptor.

Subsequently, the prepared protein and ligand structures were subjected to molecular docking and scoring using AutoDock Vina (v.1.2.0) (Eberhardt et al., 2021). Docking calculations were performed using an exhaustiveness value of 8, an energy range of 3 kcal/mol, and a maximum of 20 output poses (num_modes = 20). Docking poses were ranked according to the predicted binding affinity generated by Vina. For each receptor, the final docking pose was selected based on both the predicted binding affinity and the orientation of the PHB trimer relative to the catalytic residues to ensure a catalytically relevant binding mode.

Functional domain annotation of the candidate esterases was performed using InterProScan to identify conserved protein families, domains, and functional signatures. Domain assignments were used to evaluate the potential relationship of each candidate protein to previously characterized PHB depolymerases (Blum et al., 2026).

To further evaluate the relationship of *Te*_EPD to extracellular PHB depolymerases, multiple sequence alignment was performed using representative type 1 and type 2 PHB depolymerases. Conserved motifs characteristic of extracellular PHB depolymerases, including the lipase-box motif (GxSxG), oxyanion-hole motif (HGC), catalytic S–D–H triad, and linker regions, were examined.

Noncovalent interactions, including hydrogen bonds, identified in the molecular docking results, were analyzed using PDBsum (accessed 2 October 2025)^9^ and Discovery Studio Visualizer (BIOVIA, Dassault Systèmes, San Diego, CA, United States). All protein-ligand structures and interactions were visualized using PyMOL (The PyMOL Molecular Graphics System, v3.1, Schrödinger, LLC, New York, NY, United States).

### 2.8 Statistical analysis

All data are presented as the mean ± standard deviation (SD) of at least three independent replicates. Statistical differences were evaluated using one-way analysis of variance (ANOVA) followed by Tukey’s multiple comparison test, performed with GraphPad Prism v9.5.0 (GraphPad Software, San Diego, CA, United States). Statistical significance was defined as *p* < 0.05.

## 3 Results and discussion

### 3.1 Genomic and phylogenetic characterization of the *Terrabacter* sp. AAH1

Bacterial isolates obtained from freshwater samples collected from an agricultural stream in Gyeonggi-do, Republic of Korea (Supplementary Figure 1), were screened for PHB-degrading activity. Strain AAH1 was subcultured on an R2A agar plate containing 1% PHB emulsion to assess its extracellular PHB-degrading activity. Clear zones were observed on R2A agar plates, and the PHB film degraded even under shaking conditions in R2A broth. Therefore, Clear zone formation and film biodegradation capability were employed as primary phenotypic indicators of biodegradation efficiency.

**FIGURE 1:**
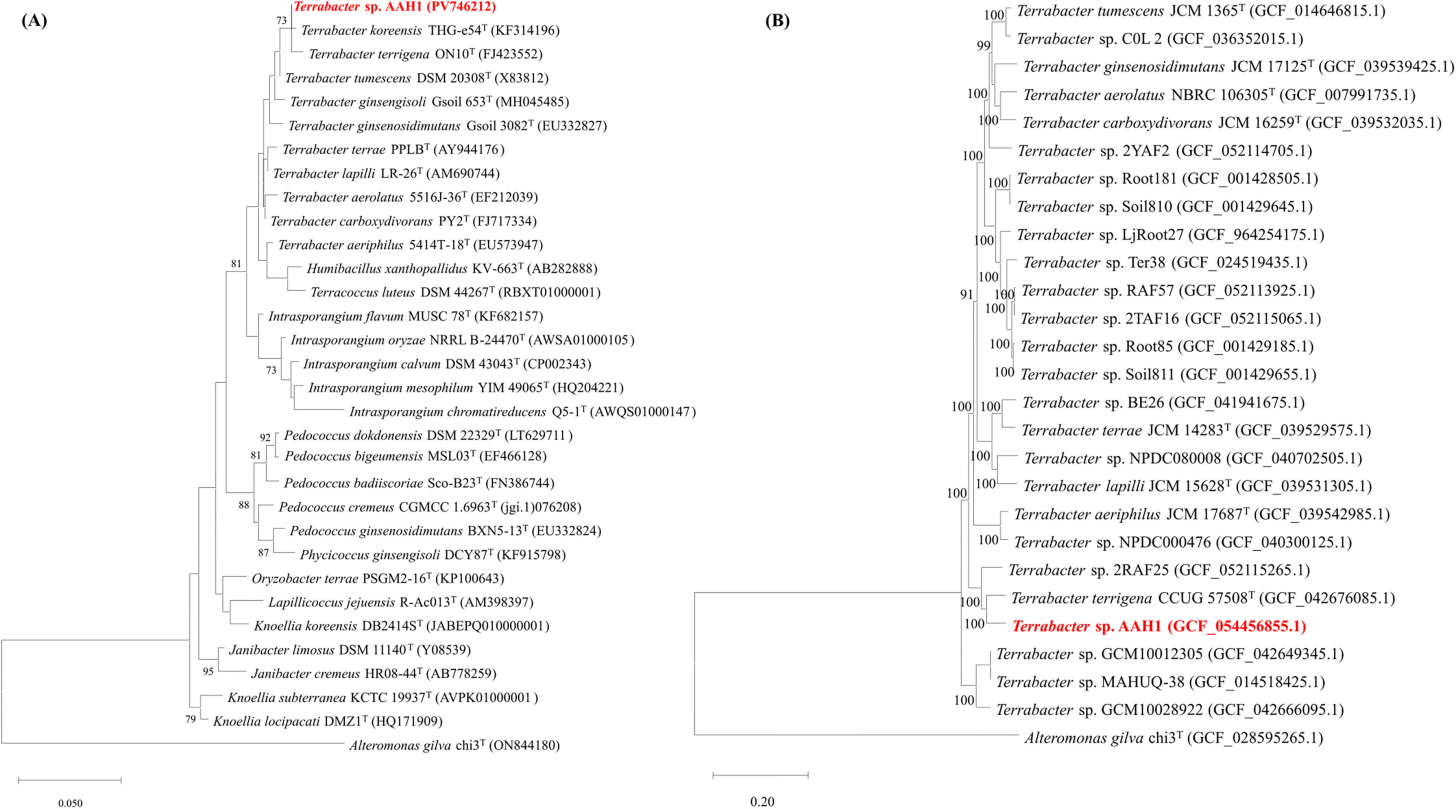
Phylogenomic placement of *Terrabacter* sp. AAH1 relative to closely related species. **(A)** Maximum-likelihood phylogenetic tree based on 16S rRNA gene sequences. Scale bars represent 0.050 substitutions per site. *Alteromonas gilva* chi3^T^ (GenBank accession no. ON844180) was used as the outgroup. **(B)** A phylogenomic tree based on 81 up-to-date bacterial single-copy core genes (UBCG2) showing the evolutionary relationships of strain AAH1. Bootstrap support values are indicated at the nodes. *A. gilva* chi3^T^ (GCF_028595265.1) was used as the outgroup. The scale bar represents 0.50 substitutions per nucleotide position.

Comparison of the 16S rRNA gene sequence of strain AAH1 against the EzBioCloud database (v.2025.04.21) and showed 99.78% similarity (1386 bp/1390 bp) to *Terrabacter koreensis* THG-e54^T^. The sequence of *Terrabacter* sp. AAH1 was deposited in the NCBI database under the accession number PV746212. Phylogenetic analysis revealed that *Terrabacter* sp. AAH1 formed a candidate monophyletic clade with *T*. *koreensis* THG-e54^T^ (99.78%), *Terrabacter terrigena* ON10^T^ (99.24%), and *Terrabacter ginsengisoil* Gsoil 653^T^ (98.61%). Further analysis encompassing 10 related genera revealed a distinct *Terrabacter* clade, supporting the unequivocal placement of strain AAH1 within the genus *Terrabacter* (Figure 1A).

To date, 10 validly published *Terrabacter* species have been isolated from diverse yet predominantly soil-related environments, including air and rocks, with eight of these species specifically reported from the Republic of Korea (Weon et al., 2007; Lee et al., 2008). In contrast, strain AAH1 was isolated from a freshwater agricultural stream, representing the first report of a *Terrabacter* strain recovered from a freshwater habitat and indicating that the genus has a broader environmental distribution than previously recognized from soil and rock-associated isolation sources.

The draft genome assembly of strain AAH1 revealed 99.96% completeness and 0% contamination, satisfying the high-quality standards required for genomic taxonomy. Phylogenomic analysis using the UBCG2 core gene set revealed that strain AAH1 clustered exclusively with *Terrabacter* species and was clearly separated from related genera, including *Intrasporangium*, *Terracoccus*, *Humibacillus*, *Pedococcus*, and *Phycicoccus* (Figure 1B).

The taxonomic position of strain AAH1 was experimentally confirmed by ANI and dDDH calculations against 25 *Terrabacter* species. Comparison with *Terrabacter terrigena* yielded an ANI of 89.56% and a dDDH value of 33.80%, while other genus members showed average values of 86.97 and 29.90%, respectively. These phylogenomic metrics, well below species-level thresholds, confirmed that strain AAH1 belongs to the *Terrabacter* species and proposed its genetic distinction from previously described species (Supplementary Figure 2). The substantial genomic divergence observed among *Terrabacter* species also reflects the considerable phylogenomic heterogeneity within this genus. Such divergence may contribute to functional variability, including the sparse distribution of putative PHB depolymerase genes identified in the present study. Consistently, core genome phylogenetic analysis based on UBCG2 supported the distinct clustering of strain AAH1, reinforcing its taxonomic independence and providing a candidate genomic framework for subsequent comparative and functional analyses. Genomic relatedness analysis was performed to confirm the taxonomic placement of strain AAH1 within the genus *Terrabacter* at the genome level, rather than to pursue formal species delineation. The ANI and dDDH values obtained against all examined *Terrabacter* species were below the proposed species-level thresholds (95% for ANI and 70% for dDDH), confirming that strain AAH1 represents a genomically distinct member of the genus *Terrabacter*. Although these values suggest potential novel species status, formal taxonomic characterization was outside the scope of the present study. Furthermore, the absence of a publicly available whole-genome sequence for the closest phylogenetic neighbor, *T. koreensis* THG-e54^T^, precluded a definitive genomic delineation in accordance with current standards for prokaryotic species description (Chun et al., 2018). Accordingly, the strain is referred to as *Terrabacter* sp. AAH1 throughout this manuscript.

### 3.2 Metabolic versatility and optimization of PHB biodegradation

Strain AAH1 exhibited a versatile extracellular enzymatic repertoire, showing positive results for protease and amylase activities through the hydrolysis of skim milk and starch, respectively. Additionally, the strain demonstrated candidate cellulase activity via the hydrolysis of carboxymethyl cellulose (Supplementary Figure 3). Notably, strain AAH1 showed no detectable lipolytic activity against Tween 20 or 80, indicating the absence of detectable extracellular lipase activity toward these substrates under the tested conditions. Given that PHB degradation was nonetheless observed, these results suggest that the PHB-degrading activity of strain AAH1 is not attributable to broad-spectrum lipolytic enzymes of the type active against Tween substrates, though the involvement of other esterase-type enzymes cannot be excluded. Morphologically, TEM analysis confirmed that AAH1 cells are non-flagellated, short rod-shaped (Supplementary Figure 4), consistent with the typical pleomorphic traits.

The PHB-degrading efficiency of strain AAH1 was highly sensitive to medium composition and environmental parameters. Among the tested media, clear zones were observed on R2A, NA, and TSA, whereas no activity was detected on MA or LB. The highest depolymerization activity was recorded in R2A, an oligotrophic (low nutrient) medium, followed by NA and TSA (Supplementary Figure 5A). Temperature assays revealed that biodegradation was maximal at 30°C, with weak clear zone formation at 25°C. No clear zone was observed at temperatures below 20°C or above 35°C (Supplementary Figure 5B). Furthermore, strain AAH1 was determined to be non-halotolerant, as growth and PHB degradation were completely inhibited under saline conditions (≥1% w/v NaCl) (Supplementary Figure 5C) on R2A agar supplemented with 0.1% (w/v) PHB emulsion. The strain exhibited PHB-degrading activity across a broad pH range (6.0–11.0), with optimal performance at pH 7.0 under the same medium conditions (Supplementary Figure 5D).

Preferential utilization of labile carbon sources significantly attenuated the PHB-degrading efficiency of strain AAH1. While PHB degradation persisted in the presence of 1% (w/v) lactose, glucose, glycerol, and sucrose, the diameter of the clear zones were significantly reduced compared to the control (Supplementary Figure 5E). No detectable chitinase activity was observed on chitin-supplemented R2A agar. Notably, the addition of fructose exerted the most potent inhibitory effect, completely abolishing clear zone formation. These results are consistent with a carbon catabolite repression (CCR)-like phenomenon, in which strain AAH1 may preferentially utilize readily available carbon sources over the complex PHB polymer. However, since no regulatory analyses were performed, this interpretation remains a working hypothesis that requires further investigation through gene expression or transcriptomic profiling to confirm the underlying regulatory mechanism.

### 3.3 Quantitative evaluation of PHB film biodegradation efficiency

Prior to evaluating biodegradation efficiency, the reliability and reproducibility of the gravimetric extraction method were confirmed through two sets of controls. PHB films subjected immediately to the extraction procedure without incubation (day 0 extraction control) yielded residual weights of 19.9, 20.0, and 20.0 mg (19.97 ± 0.06 mg) from an initial film weight of 20 mg, corresponding to a recovery rate of 99.83 ± 0.29%. Additionally, uninoculated PHB films incubated in R2A broth without strain AAH1 for the full 12-day experimental period yielded residual weights of 19.9, 19.9, and 20.0 mg (19.93 ± 0.06 mg), corresponding to a recovery rate of 99.67 ± 0.29%. These results confirm that abiotic weight loss was negligible, extraction losses were minimal, and the method demonstrated high reproducibility throughout the experimental period. To establish the optimal conditions for examining the degradative kinetics of *Terrabacter* sp. AAH1, the residual weight (mg) and resultant degradation rate (%) of the 20 mg PHB film were monitored in R2A and nutrient broth under various incubation temperatures, pH levels, and carbon sources. In accordance with the initial solid-state agar assays, the maximal PHB biodegradation activity was attained in R2A broth at 30°C. Consequently, standard R2A medium at pH 7.0 was designated as the optimal basal medium for all subsequent quantitative assays, with sterile, uninoculated media serving as negative controls to account for any potential abiotic mass loss.

**FIGURE 2:**
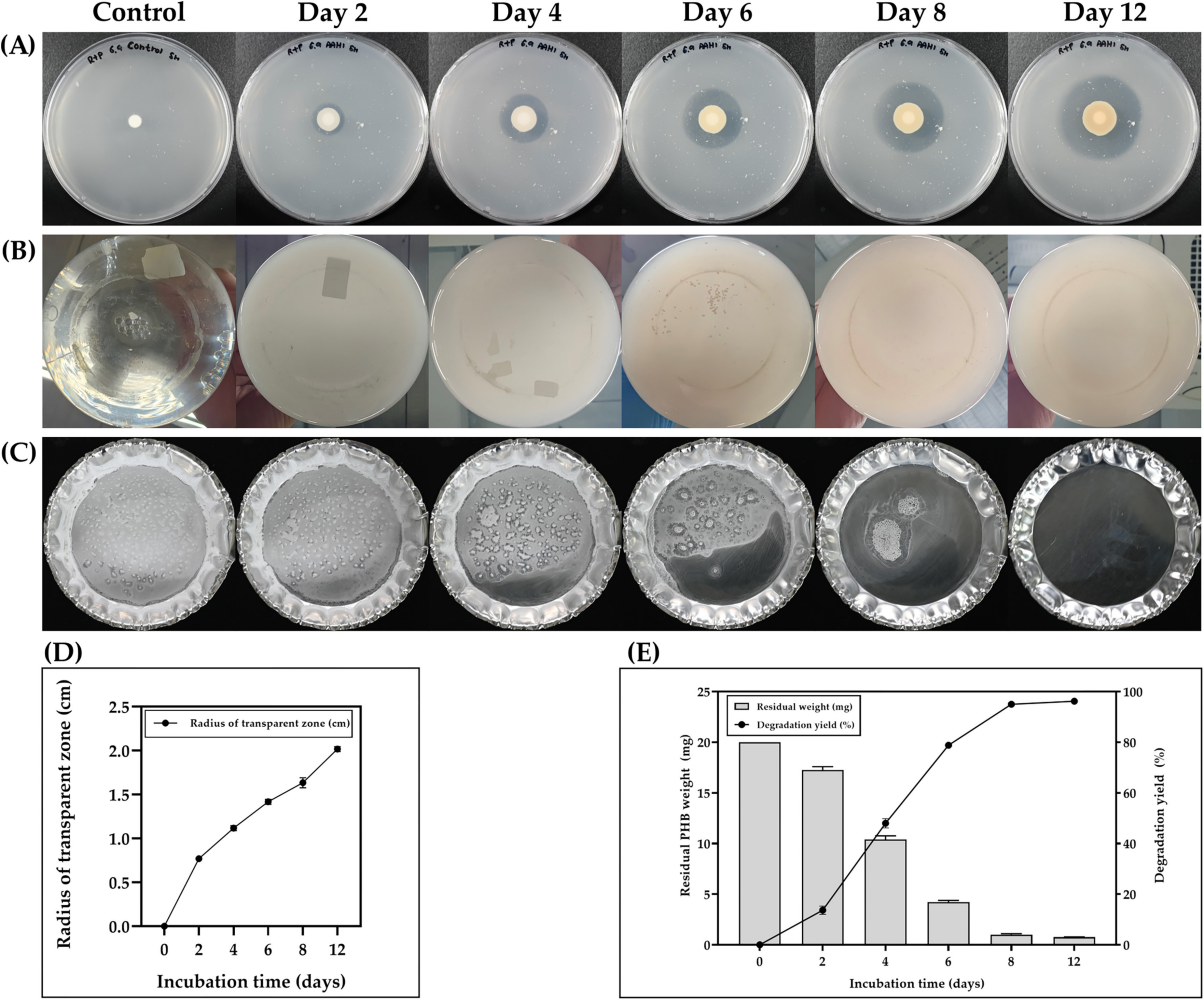
PHB degradation and yield (%) by *Terrabacter* sp. AAH1 during incubation at 30°C for 12 days. **(A)** Assessment of PHB degradation on R2A agar plates. **(B)** Assessment of PHB degradation in shake-flask cultures. **(C)** Visual observation of recovered residual PHB films. **(D)** Radius of the clear zone formed around colonies of strain AAH1. **(E)** Residual PHB film weight (mg) and degradation yield (%) following incubation with strain AAH1. Data are presented as mean ± SD of three independent replicates (*n* = 3). Statistical differences were evaluated using one-way ANOVA followed by Tukey’s multiple comparison test. Different letters above data points indicate statistically significant differences among time points (*p* < 0.05). Overall ANOVA **(D)**: *F*(5, 12) = 1,390, *p* < 0.0001; **(E)** (residual weight): *F*(5, 12) = 4,679, *p* < 0.0001.

The qualitative PHB biodegradation efficiency of strain AAH1 was first evaluated using a clear zone assay on R2A agar containing 0.1% PHB emulsion incubated at 30°C for 12 days. A clear zone of 0.77 ± 0.03 cm developed around the inoculation point on the R2A agar within 48 h. This zone exhibited a steady expansion, reaching radii of 1.12 ± 0.03, 1.42 ± 0.03, 1.63 ± 0.06, and 2.02 ± 0.03 cm at subsequent intervals (Figures 2A,D). The uninoculated control, consisting of R2A broth with a PHB film incubated under identical conditions, showed no detectable weight loss or surface morphological changes throughout the experimental period, confirming that the observed degradation was attributable solely to the biological activity of strain AAH1. In parallel, the quantitative kinetics of degradation were evaluated in a liquid-phase shaking culture using 50 mL of R2A broth with a 20 mg PHB film. In the liquid-phase assay, no discernible surface changes were detected after 2 days of incubation, although a measurable reduction in residual weight (17.27 ± 0.32 mg) corresponding to a biodegradation rate of 13.67 ± 1.61 % was recorded. The degradative process underwent rapid acceleration from day 4 onward, paralleling the onset of film fragmentation. The mean residual weight decreased precipitously from 10.40 ± 0.36 mg (48.00 ± 1.80%) on day 4 to near-complete degradation of 0.40 ± 0.02 mg (98.02 ± 0.08%) by day 12, with transient values of 4.23 ± 0.15 mg (78.83 ± 0.76%) and 1.00 ± 0.10 mg (95.00 ± 0.50%) on days 6 and 8, respectively (Figures 2B,C,E).

This fragmentation process intensified progressively over time, facilitating a high-efficiency degradative turnover. A marginal deceleration in the degradation rate was observed after day 8, which might be attributed to the depletion of labile nutrients in the oligotrophic R2A medium or the accumulation of metabolic by-products that potentially altered the local microenvironment. To our knowledge, this is the first report to provide empirical evidence of PHB biodegradation within the genus *Terrabacter*, suggesting a potential ecological role in bioplastic degradation within freshwater environments.

### 3.4 Morphological and structural analysis of degraded PHB films

**FIGURE 3:**
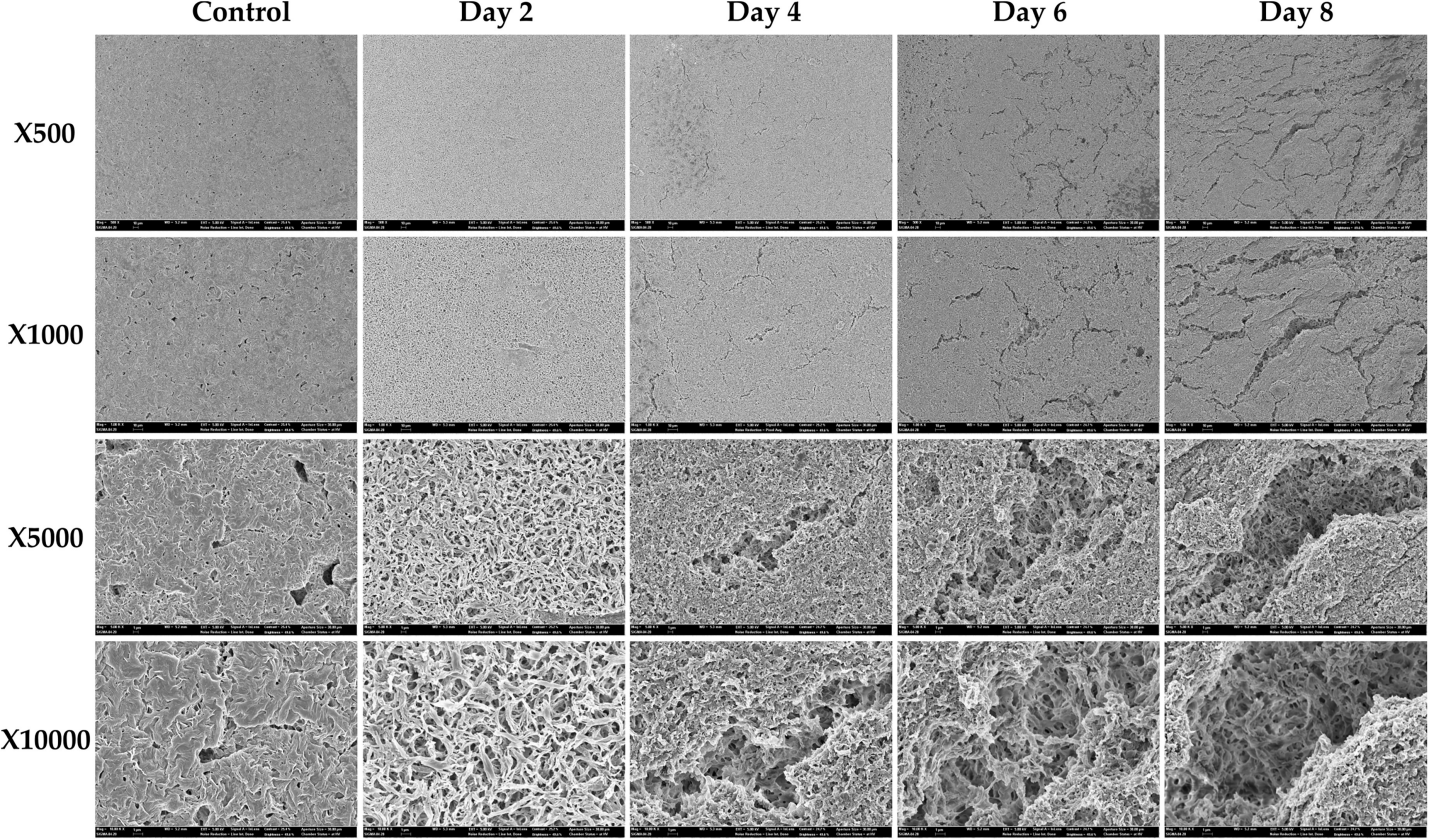
Morphological characterization of PHB degradation. SEM image showing surface morphological changes of a PHB film biodegraded by *Terrabacter* sp. AAH1 characterized by the formation of pores and surface cracks during degradation.

SEM analysis revealed the biodegradation mechanism of strain AAH1 by visualizing the progressive morphological transformation of the PHB films (Figure 3). The control film exhibited a predominantly pristine and homogenous surface, punctuated only by subtle manufacturing-related textures, while maintaining full structural integrity. Upon incubation with strain AAH1, distinct biodegradation phases were observed. Initial exposure to the isolate triggered a transition to diffuse surface erosion by day 2, rather than localized, discrete pitting. This etched morphology is consistent with enzymatic surface erosion, possibly involving the action of secreted hydrolytic enzymes, though the specific mechanism cannot be determined from morphological data alone. A critical structural threshold was reached by day 4, evidenced by the emergence of extensive micro-cracks. This phenomenon implies that the enzymatic hydrolysis of the polymer chains induced material embrittlement and a concomitant loss of tensile strength. By days 6 and 8, these cracks propagated significantly. The surface appeared highly porous with compromised architecture, suggesting that the biodegradation process may involve both surface erosion and subsequent bulk disintegration. This physical breakdown drastically increased the surface area accessible for enzymatic activity, thereby potentially increasing the surface area accessible for enzymatic activity.

**FIGURE 4:**
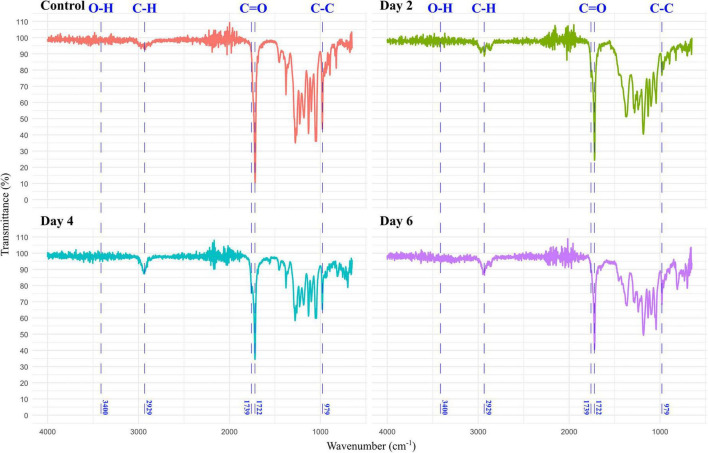
Chemical characterization of PHB degradation. FT-IR spectral analysis illustrating time-dependent changes in functional groups of the PHB film during biodegradation by *Terrabacter* sp. AAH1 at 30°C. X-axis: wavenumber (cm^– 1^); Y-axis: transmittance (%).

Changes in the chemical structure of the PHB film were monitored via FT-IR analysis at 2-day intervals over a 6-day incubation period (Figure 4). Consistent with the gravimetric data, films sampled after Day 8 were excluded from spectroscopic analysis as the high degradative turnover led to complete structural collapse and fragmentation, rendering subsequent measurements unfeasible.

Progressive changes in transmittance were observed across multiple characteristic bands during the incubation period. Increases in transmittance were observed for the crystalline ester carbonyl band at 1,722 cm^–1^ (27.13–45.20%) and the amorphous ester carbonyl band at 1,739 cm^–1^ (62.39–76.60%) by day 6, which may be consistent with ester bond cleavage during polymer backbone hydrolysis, though changes in film thickness and surface roughness may also contribute to these spectral shifts (Kann et al., 2014). Concurrently, a decrease in transmittance from 98.45 to 96.59% was observed in the broad O-H stretching vibration band at 3,400 cm^–1^, which could reflect the accumulation of hydroxyl-terminated chain cleavage products, although surface roughening and increased light scattering associated with film degradation may also account for this change (Kim et al., 2023). A notable increase in transmittance from 46.73 to 76.82% was observed within the first 48 h for the C-C stretching vibration at 979 cm^–1^, which may be associated with disruption of the crystalline lattice during the initial biodegradation stage, though alternative explanations including film thinning cannot be excluded. Furthermore, a decrease in transmittance from 93.37 to 88.76% was observed for the asymmetric methyl stretching band at 2,929 cm^−1^, which has been suggested to reflect physical surface erosion rather than changes in copolymer composition (Adar et al., 2023), though this interpretation should be treated with caution as surface roughness effects may also contribute to the observed signal changes.

Taken together, the FT-IR spectral changes observed across multiple characteristic bands are consistent with progressive chemical and structural alterations in the PHB film during biodegradation by strain AAH1. However, it should be noted that FT-IR transmittance changes in degrading polymer films can reflect a combination of chemical changes, film thickness reduction, and surface roughness effects, and the relative contributions of these factors cannot be fully decoupled from the present data alone.

This increased signal intensity likely resulted from surface roughening and the exposure of methyl-rich terminal groups generated during enzymatic chain scission. SEM and FT-IR analyses confirmed that *Terrabacter* sp. AAH1 induced profound and systematic physical and chemical transformations in the PHB film.

### 3.5 Functional genomic profiling of the PHB biodegradation pathway

**FIGURE 5:**
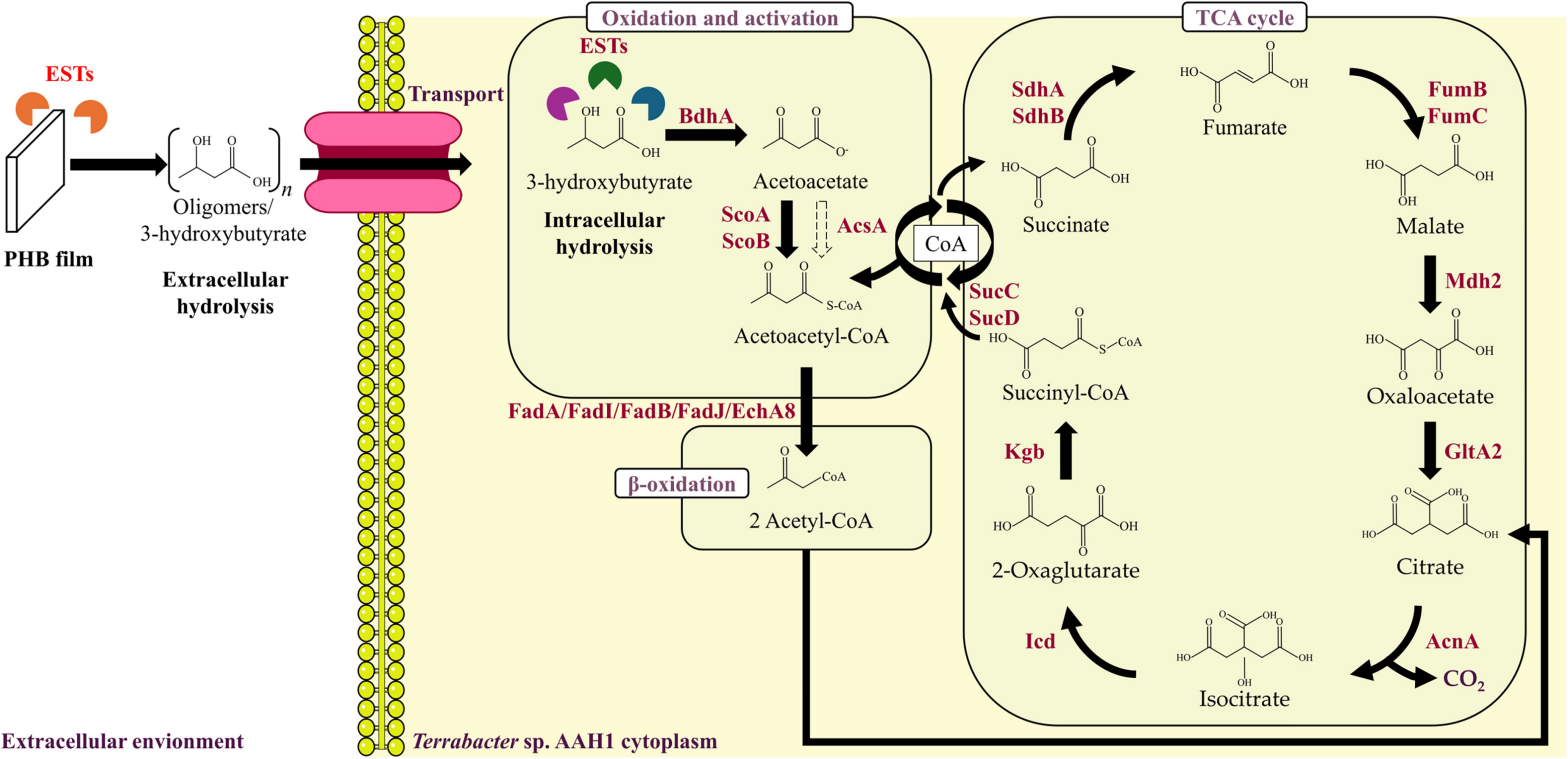
Predicted metabolic pathway for PHB degradation in *Terrabacter* sp. AAH1. This schematic illustrates a hypothetical model for PHB degradation in *Terrabacter* sp. AAH1, based on *in silico* predictions including signal peptide analysis, structural modeling, and molecular docking simulations. ESTs denote putative esterase-family proteins, including *Te*_EPD (orange), *Te*_AES2 (green), *Te*_AES1 (purple), and *Te*_YbfF (blue).

The observed clear zone formation on PHB-containing agar plates and the quantitative degradation of PHB films in liquid culture provided phenotypic evidence for extracellular PHB-hydrolyzing activity in strain AAH1. Since PHB is an insoluble solid polymer that cannot be directly transported across the cell membrane, its initial hydrolysis necessarily requires extracellular enzymatic activity. Based on this phenotypic evidence, genome-wide screening was conducted to identify putative extracellular esterase candidates. The complete genome of strain AAH1 comprises 4,644,444 bp with a G+C content of 71.5 mol%. It encodes a full suite of RNA genes, including 53 tRNAs. Among the 4,281 predicted functional coding sequences (CDSs), classified across 26 eggNOG categories, we identified 27 genes predicted to be involved in PHB depolymerization and subsequent degradation. The symmetrical patterns of G+C content and GC skew substantiate the high integrity of the genome assembly (Supplementary Figure 6). To ensure stringent functional annotation, only genes exhibiting as E-value ≤ 1 × 10^–50^ were selected for metabolic reconstruction (Supplementary Excel File 1). Based on these rigorous criteria, the genetic repertoire involved in PHB catabolism was categorized into four functional modules: PHB depolymerization and oligomer hydrolysis, oxidation and activation, β-oxidation-like pathway, and TCA cycle and energy metabolism (Figure 5 and Supplementary Table 1). Genome-wide screening identified four putative esterase-family genes (*Te*_EPD, *Te*_YbfF, *Te*_AES1, and *Te*_AES2) as primary candidates for PHB hydrolysis. For the subsequent oxidation and activation of monomeric 3-HB, the genome encodes 3-HB dehydrogenase (*bdhA*) and a putative succinyl-CoA:3-ketoacid CoA transferase system (*scoA*, *scoB*) (Corthésy-Theulaz et al., 1997), which is predicted to facilitate the conversion of acetoacetate to acetoacetyl-CoA via a CoA-transfer mechanism analogous to the well-characterized SCOT pathway. This pathway is putatively complemented by multiple paralogs of ATP-dependent acetyl-CoA synthetase (*acsA*_1, *acsA*_2, *acsA*_3) (Lindenkamp et al., 2013), which exhibit broad substrate specificity for short-chain carboxylic acids and may serve as an alternative, ATP-dependent activation route under specific metabolic conditions (Volodina et al., 2016). The downstream β-oxidation-like pathway is supported by a comprehensive gene set, including 3-ketoacyl-CoA thiolase (*fadA*, *fadI*), hydroxyacyl-CoA dehydrogenase (*fadB*, *fadJ*_1, *fadJ*_2), and enoyl-CoA hydratase (*echA8*), which together mediate the thiolytic cleavage of acetoacetyl-CoA to yield two molecules of acetyl-CoA. Furthermore, the presence of core TCA cycle enzymes (*gltA2*, *acnA*, *icd*, *kgd*, *sucC*, *sucD*, *sdhA*, *sdhB*, *fumB*, *fumC*, *mdh*_2) suggests the strain’s capacity for efficient energy production and carbon assimilation from PHB-derived intermediates. Notably, the succinyl-CoA regenerated through the TCA cycle via *sucC*/*sucD* is proposed to serve as the CoA donor for the *scoA*/*scoB*-mediated activation of acetoacetate, thereby suggesting a potential metabolic link between the TCA cycle and the upstream activation step. Collectively, these findings suggest that *Terrabacter* sp. AAH1 may harbor a genomically encoded metabolic framework potentially capable of catabolizing PHB-derived intermediates, though experimental confirmation of individual enzymatic steps remains necessary. The predicted catabolic architecture in strain AAH1 comprising putative extracellular hydrolysis, intracellular CoA-mediated activation of 3-HB-derived intermediates, β-oxidation-like chain processing, and entry into the TCA cycle is consistent with the experimentally validated PHA catabolic pathway recently described in the marine bacterium *Alteromonas plasticoclasticus* MED1, in which combined genomic and transcriptomic analyses confirmed coordinated activation of analogous catabolic modules during PHBV biodegradation (Barbe et al., 2024). The putative extracellular depolymerase (originally annotated as a hypothetical protein; BLOJFJFB_02657) was designated as *Te*_EPD based on its classification within the e_dPHAscl_type1 family by InterProScan and its sequence homology to biochemically validated extracellular PHB depolymerases (Viljakainen and Hug, 2021), while the remaining esterases were named *Te_*YbfF, *Te_*AES1, and *Te_*AES2 based on their predicted structural domains and hydrolytic roles.

**FIGURE 6:**
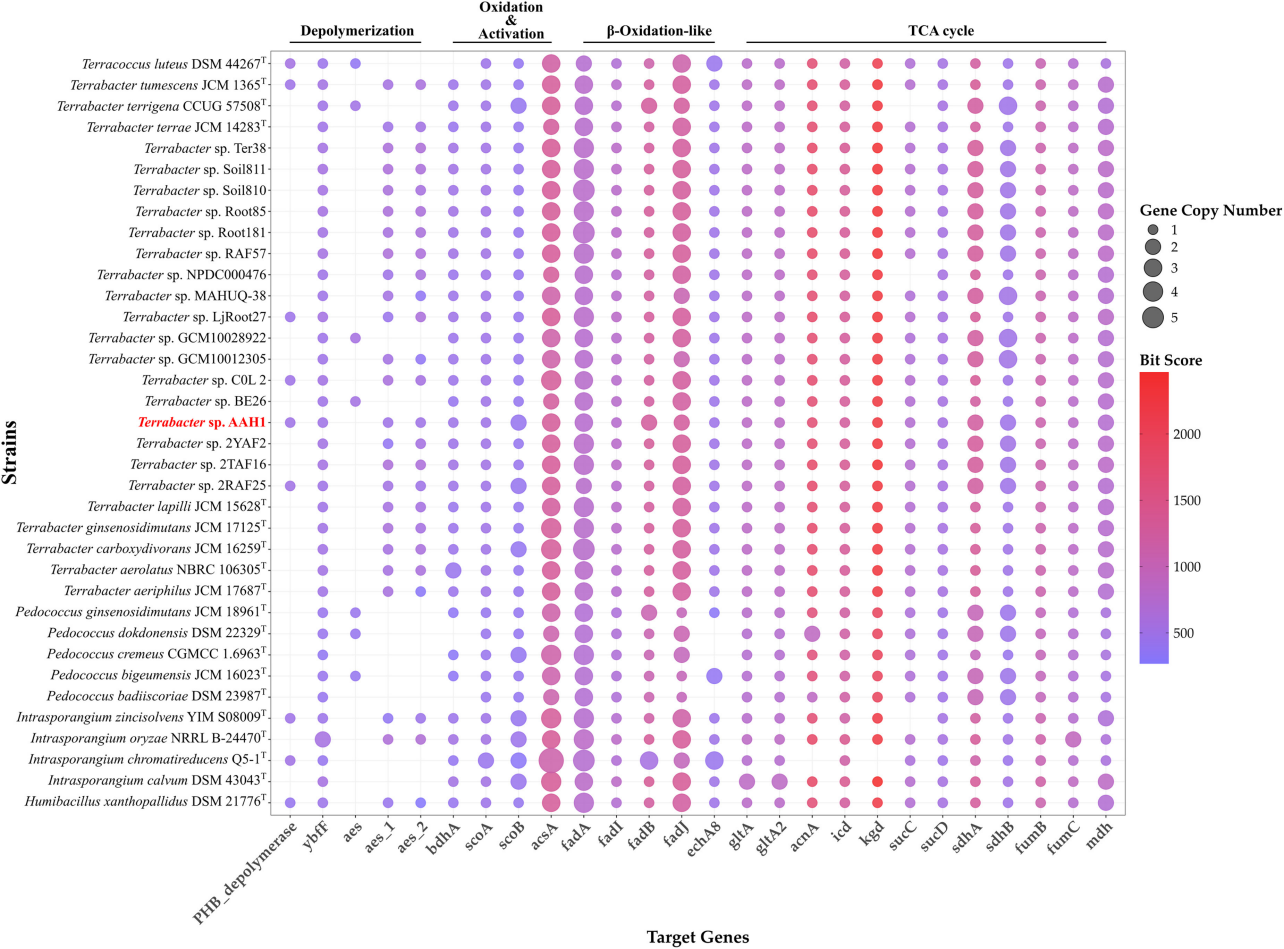
Comparative genomic distribution and conservation of key genes associated with PHB biodegradation and downstream metabolism. The bubble heatmap illustrates the genomic prevalence, copy number, and sequence homology of target genes across 25 *Terrabacter* species and 11 related strains (*Pedococcus*, *Intrasporangium*, *Terracoccus*, and *Humibacillus*). The target genes are categorized into four functional modules: depolymerization, oxidation and activation, β-oxidation-like pathway, and the TCA cycle. The size of each bubble corresponds to the gene copy number within the respective genome, while the color gradient represents the degree of sequence homology expressed as the bit score.

Of the 37 genomes evaluated, 36 exhibited completeness values ≥ 98.82% and contamination levels < 2%, satisfying the high-quality standards required for comparative analysis (Supplementary Table 2). *Intrasporangium flavum* MUSC 78^T^ (GCF_001956915.1) was excluded from subsequent comparative analysis due to a completeness value of 81.31%, which may result in underrepresentation of genes and potentially confound comparative assessments. Accordingly, comparative genomic analysis was performed across a final set of 36 genomes. To gain deeper insights into the genetic mechanisms underlying the superior PHB-degrading phenotype of strain AAH1 and to investigate the evolutionary prevalence of these traits within the genus *Terrabacter*, a comparative genomic analysis was performed. Comparative analysis of 27 key genes across 25 *Terrabacter* species and 11 related taxa (*Pedococcus*, *Intrasporangium*, *Terracoccus*, and *Humibacillus*) revealed a highly restricted distribution of putative extracellular PHB depolymerases (Figure 6). Among the 25 *Terrabacter* species examined, only five specifically strain AAH1, JCM 1365^T^, LjRoot27, C0L2, and 2RAF25 harbored a putative PHB depolymerase homolog. A comparable pattern was observed in *Intrasporangium* (2/4 genomes), while no depolymerase homolog was detected in any of the five *Pedococcus* genomes examined. However, the apparent absence of depolymerase genes in certain taxa should be interpreted with caution. The putative PHB depolymerase homologs detected across *Terrabacter* and related genera exhibited relatively low bit scores (range: 399–534), indicating considerable sequence divergence from *Te*_EPD of strain AAH1. This divergence raises the possibility that some detected homologs may represent functionally distinct esterases rather than experimentally validated PHB depolymerases, and conversely, that additional divergent depolymerase sequences may have evaded detection in genomes where no homolog was identified. Accordingly, annotation limitations, sequence divergence, and differences in genome completeness should be considered when interpreting the comparative genomic distribution of putative PHB depolymerase genes. Furthermore, the genomic presence of a depolymerase homolog does not necessarily confer extracellular functionality, as evidenced by the contrasting signal peptide predictions for strain AAH1 (Sec-type, probability: 0.9984) and *Terrabacter* sp. LjRoot27 (intracellular, probability: 1,000) discussed in the following section. It should be noted that signal peptide prediction tools including SignalP 6.0 are not infallible, and localization assays or secretome analyses would be required to experimentally confirm the extracellular function of *Te*_EPD. In contrast to the restricted distribution of PHB depolymerase genes, intracellular enzymes involved in downstream PHB catabolism demonstrated markedly higher conservation. Genes encoding the CoA-transferase system (*scoA*, *scoB*), core β-oxidation enzymes (*fadA*, *fadI*, *fadB*, *fadJ*, *echA8*), and TCA cycle components (*gltA2*, *acnA*, *icd*, *kgd*, *sucC*, *sucD*, *sdhA*, *sdhB*, *fumB*, *fumC*, *mdh*) were detected in all or nearly all *Terrabacter* genomes with consistently high bit scores (mean bit score range: 402–2,313), indicating strong sequence conservation across the genus. Notably, *acsA* homologs were universally present across all examined genera, including *Pedococcus*, *Intrasporangium*, *Terracoccus*, and *Humibacillus* (mean bit score > 1,100 across all genera), suggesting that the capacity for CoA-dependent carboxylate activation represents a broadly conserved metabolic trait among these Actinobacteria. Regarding intracellular esterases, *ybfF* homologs were conserved across all examined genera (25/25 in *Terrabacter*; 5/5 in *Pedococcus*). While *aes*_1 and *aes*_2 homologs were absent from all *Pedococcus* genomes and from two of four *Intrasporangium* genomes, *aes* homologs were detected in three of five *Pedococcus* species, indicating that a basal esterase repertoire is conserved in this genus despite the absence of certain paralog subgroups. Collectively, these findings suggest that the putative extracellular PHB depolymerase represents a specialized genomic adaptation rather than an ancestral trait of the genus *Terrabacter*, potentially acquired through niche-specific evolutionary pressures. The co-occurrence of a phylogenetically restricted putative extracellular depolymerase with broadly conserved downstream catabolic genes may contribute to the efficient PHB-degrading phenotype observed in strain AAH1, though a causal relationship remains to be experimentally demonstrated.

*In silico* secretion profiling using SignalP 6.0 provided critical functional differentiation between the predicted depolymerases. Strain AAH1’s *Te*_EPD harbored a high-confidence Sec-type signal peptide (probability: 0.9984), strongly suggesting its putative extracellular localization. In contrast, the depolymerase ortholog in *Terrabacter* sp. LjRoot27 was predicted to be intracellular (probability: 1,000). This distinction highlights that the mere genotypic presence of a depolymerase gene does not necessitate an extracellular degradation phenotype. This genetic architecture may enable AAH1 to go beyond initial polymer hydrolysis, potentially integrating degradation products into central metabolism, which suggests a putative role in bioplastic degradation within freshwater ecosystems.

### 3.6 Computational modeling and molecular docking

Molecular docking of the PHB trimer substrate was performed using AutoDock Vina to assess substrate binding across the four putative PHB depolymerase models. Docking grids were individually defined for each receptor based on substrate-binding pockets predicted by Fpocket (Supplementary Figure 7 and Supplementary Table 3).

Three-dimensional structures of four putative PHB depolymerase homologs—*Te*_EPD, *Te*_YbfF, *Te*_AES1, and *Te*_AES2—were modeled using ColabFold. The predicted models showed high overall confidence, with most residues displaying predicted Local Distance Difference Test (pLDDT) scores above 70, especially in the conserved catalytic regions (Supplementary Figures 7B,F,J,N). Low-confidence regions were primarily located in surface-exposed loops distant from predicted binding sites and catalytic residues; therefore, they were unlikely to influence substrate binding but were retained in the docking models.

Multiple sequence alignment and structural alignment were performed to evaluate sequence and structural conservation of each modeled protein using homologous esterases identified by BLAST analysis. Homologs were chosen for high sequence similarity and availability of experimentally determined three-dimensional structures. Accordingly, *Te*_EPD was aligned with *Aspergillus sydowii* feruloyl esterase (*AS*_FeaE, PDB ID: 8IY8) (Phienluphon et al., 2023) and *Aspergillus awamori* acetylxylan esterase (*AL*_AXE, PDB ID: 5X6S) (Komiya et al., 2017); *Te*_YbfF with *Escherichia coli* esterase YbfF (*E. coli*_YbfF, PDB ID: 3BF8) (Park et al., 2008) and East Pacific Rise bacterium putative esterase (EprEST, PDB ID: 7C4D) (Zhu et al., 2021); *Te*_AES1 with *Parvibaculum* EST8 (*Pv*_Est8, PDB ID: 4YPV) (Pereira et al., 2017) and *Pyrobaculum calidifontis* esterase (*PC*_EST, PDB ID: 2YH2) (Palm et al., 2011); and *Te*_AES2 with *Escherichia coli* acetyl esterase (*E. coli*_AES, PDB ID: 4KRX) (Schiefner et al., 2014) and *PC*_EST. Multiple sequence alignment revealed high conservation of residues corresponding to the S–D–H catalytic triad across each model and its respective homologs (Supplementary Figures 7A,E,I,M). This sequence-level conservation was consistent with the conserved spatial positioning of the S–D–H catalytic triad observed in the structural superposition of the active sites (Supplementary Figures 7D,H,L,P). Structural alignment further demonstrated a highly conserved overall fold and active-site architecture across all comparisons (Supplementary Figures 7C,G,K,O). Consistent with these observations, pairwise structural superpositions yielded RMSD values ranging from 0.644 to 1.643 Å, indicating a high degree of structural similarity between the modeled proteins and their homologous esterases (Supplementary Table 4).

Molecular docking of the PHB trimer substrate was performed using AutoDock Vina to assess substrate binding across the four esterase candidates. Since the homologous esterases used for structural comparison do not bind PHB, a previously characterized PHB depolymerase from *Talaromyces funiculosus* (PDB ID: 2D81) (Hisano et al., 2006) was included as a positive control to provide a reference PHB binding affinity. The control PHB depolymerase exhibited a binding affinity of –5.6 kcal/mol. In all four models, the PHB trimer was preferentially accommodated within the predicted active-site pocket, yielding binding affinities of –4.9 kcal/mol for *Te*_EPD, –6.8 kcal/mol for *Te*_YbfF, –6.1 kcal/mol for *Te*_AES1, and –5.5 kcal/mol for *Te*_AES2 (Supplementary Table 5). These affinities were comparable to or stronger than that of the control PHB depolymerase, indicating that all four esterase candidates possess substrate-binding characteristics compatible with PHB recognition.

**FIGURE 7:**
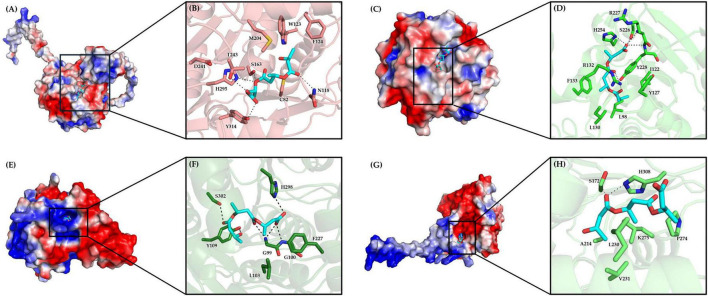
Molecular docking of a PHB trimer with putative PHB depolymerases. PHB trimer molecules are shown as cyan sticks. **(A,C,E,G)** Electrostatic surface diagram of the putative PHB depolymerases *Te*_EPD, *Te*_YbfF, *Te*_AES1, and *Te*_AES2, showing the docked PHB trimer within the predicted substrate-binding pocket. Negatively and positively charged surface regions are shown in red and blue, respectively. **(B,D,F,H)** Close-up views of PHB trimer binding sites. Key interacting residues are shown as sticks; the PHB trimer as sticks, and hydrogen bonds by dashed lines.

The conserved Ser–Asp–His (S–D–H) catalytic triad identified in all four putative PHB depolymerase models is consistent with the canonical catalytic machinery of serine esterases (Knoll et al., 2009). In this triad, the serine residue is positioned to act as the nucleophile that directly attacks the ester bond of the PHB substrate, while the histidine residue functions as a general base to facilitate serine activation. The aspartate residue, or in some esterases a glutamate residue, stabilizes the protonated histidine through electrostatic interactions. Both aspartate and glutamate possess negatively charged carboxylate side chains, enabling effective modulation of histidine protonation during catalysis. The conserved spatial arrangement of the S–D–H catalytic triad observed in structural alignment, together with the consistent orientation of the PHB ester backbone toward this triad in the docking simulations, supports the functional relevance of this catalytic architecture in the modeled proteins (Figure 7).

In addition to the conserved catalytic features, *Te*_EPD and *Te*_AES2 exhibited an additional N-terminal sequence extension that was not observed in *Te*_YbfF or *Te*_AES1 (Supplementary Figures 7B,N). This N-terminal region displays characteristics commonly associated with signal peptides, including an extended hydrophobic segment. Notably, in the case of *Te*_EPD, homologous proteins reported in previous studies contain experimentally or computationally annotated signal peptides in the corresponding N-terminal region. Based on these sequence features and homology-based observations, *Te*_EPD and *Te*_AES2 may potentially function as secreted enzymes, which would be consistent with a role in extracellular PHB biodegradation.

Collectively, these analyses indicate that all four candidate proteins possess conserved esterase-like catalytic architectures and exhibit substrate-binding characteristics compatible with PHB recognition. However, these features are not sufficient to distinguish experimentally validated PHB depolymerases from other esterases. Therefore, additional analyses were performed using experimentally characterized extracellular PHB depolymerases.

To further distinguish potential PHB depolymerases from general esterases, additional sequence comparisons were performed using experimentally characterized extracellular PHB depolymerases. Because strain AAH1 exhibited clear zone formation on PHB-containing agar and efficiently degraded PHB films in liquid culture, comparative analyses focused on extracellular PHB depolymerases responsible for the initial hydrolysis of environmental PHB polymers. In contrast, intracellular PHB depolymerases primarily function in the mobilization of intracellular PHB storage granules and were therefore not considered primary candidates for the extracellular PHB-degrading phenotype observed in this study. Functional domain analysis using InterProScan revealed clear differences among the candidate esterases. *Te*_EPD was assigned to the extracellular short-chain-length polyhydroxyalkanoate depolymerase type 1 (e_dPHAscl_type1) family and contained a conserved α/β-hydrolase catalytic domain. In contrast, *Te*_AES2 was annotated only as a member of the AB hydrolase superfamily and α/β-hydrolase family without specific association to known PHB depolymerases (Table 1).

**TABLE 1:** Functional domain annotation and signal peptide prediction of the esterase candidates identified in *Terrabacter* sp. AAH1.

Protein	InterPro annotation	Predicted function
*Te*_EPD	e_dPHAscl_type1 family; α/β hydrolase domain	Putative Type 1 extracellular PHB depolymerase
*Te*_AES2	AB hydrolase superfamily; α/β hydrolase domain;	Putative intracellular esterase

**FIGURE 8:**
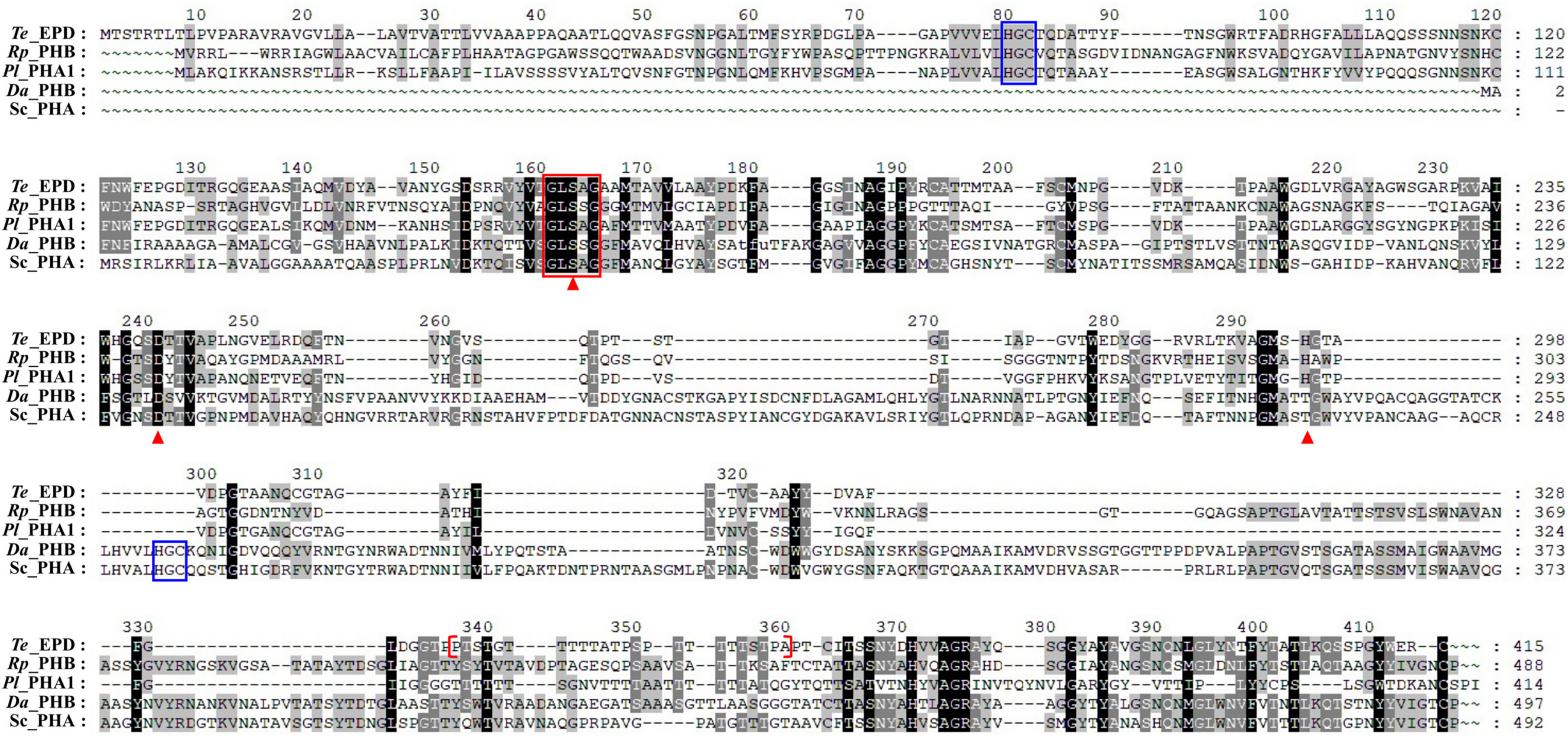
Multiple sequence alignment of *Te*_EPD with representative extracellular PHB depolymerases. Multiple sequence alignment was performed using *Te*_EPD and representative type 1 PHB depolymerases from *Ralstonia pickettii* (*Rp*_PHB) and *Paucimonas lemoignei* (*Pl*_PHA1), together with representative type 2 PHB depolymerases from *Delftia acidovorans* (*Da*_PHB) and *Schlegelella* sp. KB1a (*Sc*_PHA). Conserved oxyanion-hole motifs (HGC) are indicated by blue boxes, and conserved lipase-box motifs (GxSxG) are indicated by red boxes. Residues corresponding to the catalytic Ser–Asp–His (S–D–H) triad are marked with red triangles. The threonine-rich linker region identified in *Te*_EPD is indicated by a red bracket.

To further evaluate the relationship of *Te*_EPD to experimentally characterized extracellular PHB depolymerases, multiple sequence alignment was performed using representative type 1 PHB depolymerases compiled from the biochemically validated reference set described by Viljakainen and Hug (2021), including type 1 seqeunces from *Ralstonia pickettii* (*Rp*_PHB) (Saito et al., 1989), *Paucimonas lemoignei* (*Pl*_PHA1) (Jendrossek et al., 1993), and type 2 sequences from *Delftia acidovorans* (*Da*_PHB) (Kasuya et al., 1997), and *Schlegelella* sp. KB1a (*Sc*_PHA) (Romen et al., 2004), classified according to catalytic motif organization (Jendrossek and Handrick, 2002; Figure 8).

The analysis revealed conservation of the catalytic lipase-box motif (GxSxG) across *Te*_EPD and all reference depolymerases. Notably, the putative oxyanion-hole motif (HGC) of *Te*_EPD was located upstream of the lipase-box motif, consistent with the arrangement observed in type 1 PHB depolymerases. In contrast, representative type 2 PHB depolymerases possessed the HGC motif downstream of the lipase-box motif. Furthermore, the catalytic S–D–H triad of *Te*_EPD was conserved and aligned with the corresponding catalytic residues of type 1 PHB depolymerases. Analysis of the substrate-binding region further revealed the presence of a threonine-rich linker region in *Te*_EPD. Although the amino acid sequence of this linker differed from those of the reference type 1 depolymerases included in this study, threonine-rich linker regions are commonly reported among extracellular PHB depolymerases and are thought to contribute to substrate interaction and enzyme flexibility. In addition, *Te*_EPD contains an approximately C-terminal region downstream of the linker. Although this region was not annotated as a recognizable substrate-binding domain (SBD) by InterProScan and did not exhibit significant sequence similarity to previously characterized PHB depolymerase SBDs, its position within the protein architecture is consistent with a potential role in substrate interaction. Collectively, these sequence features indicate that *Te*_EPD most closely resembles type 1 extracellular PHB depolymerases.

These observations suggest that *Te*_EPD is the most likely candidate to function as a experimentally validated PHB depolymerase, whereas *Te*_YbfF, *Te*_AES1, and *Te*_AES2 may instead participate in downstream hydrolysis or metabolism of PHB degradation products. Although experimental validation will be required to confirm these functional assignments, the combined sequence, structural, and docking analyses support distinct roles for these esterases in the PHB biodegradation pathway.

## 4 Conclusion

This study provides the first experimental evidence of highly efficient PHB biodegradation at the strain level by the newly isolated *Terrabacter* sp. AAH1. The genomic and structural basis of this phenotype was further investigated through comparative genomic analysis and *in silico* structural modeling, which identified putative enzymatic candidates for PHB hydrolysis, though biochemical validation of these proposed functions remains to be performed. Under laboratory conditions, strain AAH1 achieved a remarkable PHB weight loss of 98.02 ± 0.08% within only 12 days. SEM and FT-IR analyses provided complementary physicochemical evidence supporting the gravimetric results.

Comparative genomic analysis indicated that putative extracellular PHB depolymerase genes are distributed in a phylogenetically restricted manner within the genus *Terrabacter*, whereas genes encoding intracellular enzymes involved in downstream PHB catabolism were detected across the majority of examined genomes. The genomic co-occurrence of a putative Sec-type secreted depolymerase and a predicted catabolic gene complement in strain AAH1 is consistent with the hypothesis that both extracellular hydrolysis and intracellular assimilation of PHB-derived intermediates may be genomically supported in this strain. Collectively, these findings suggest that the capacity for extracellular PHB depolymerization may constitute a lineage-specific genomic trait within *Terrabacter*, acquired independently of the broadly conserved downstream metabolic machinery.

Building upon this genomic architecture, *in silico* structural modeling and molecular docking analyses were performed for four candidate esterases identified in the AAH1 genome. Although all four proteins *Te*_EPD, *Te*_YbfF, *Te*_AES1, and *Te*_AES2 harbor a conserved S–D–H catalytic triad and exhibited predicted binding affinities for the PHB trimer comparable to or exceeding that of a reference PHB depolymerase, the conservation of this triad alone is insufficient to distinguish bona fide PHB depolymerases from general esterases, as it is broadly shared across the α/β-hydrolase superfamily. Functional differentiation among the four candidates was therefore based on additional criteria, including InterProScan domain classification, signal peptide prediction, and conserved motif architecture. Among the four candidates, *Te*_EPD was uniquely assigned to the extracellular short-chain-length PHA depolymerase type 1 (e_dPHAscl_type1) family, harbored a type 1-specific HGC oxyanion hole motif upstream of the lipase box, and carried a high-confidence Sec-type signal peptide, collectively supporting its designation as the primary putative extracellular PHB depolymerase. In contrast, *Te*_YbfF, *Te*_AES1, and *Te*_AES2 lacked signal peptides and did not exhibit domain architectures consistent with characterized extracellular PHB depolymerases, suggesting putative intracellular roles in the hydrolysis of soluble PHB-derived oligomers or related metabolic intermediates. Taken together, these *in silico* analyses are consistent with a hypothetical model in which extracellular depolymerization of PHB is initiated by *Te*_EPD, with subsequent intracellular processing potentially involving the remaining esterase candidates, though biochemical and localization studies will be required to validate this proposed functional partitioning.

Collectively, these results position strain AAH1 as a genomically and functionally distinct PHB degrader with significant ecological relevance for freshwater bioplastic degradation. The experimentally demonstrated degradation efficiency (98.02 ± 0.08% weight loss within 12 days), together with *in silico* evidence suggesting PHB-binding capability of the putative *Te*_EPD enzyme, supports further investigation into the depolymerase activity of strain AAH1. From a biotechnological perspective, the specialized genomic architecture of AAH1 provides a candidate genetic resource warranting further functional characterization for metabolic engineering and the development of specialized systems for rapid plastic waste treatment. These findings provide a genomic and *in silico* basis for future investigations into the PHB-degrading potential of *Terrabacter* sp. AAH1 and may inform the development of microbial strategies for bioplastic waste management. Future studies should focus on biochemical characterization and high-resolution structural determination to further validate and optimize the catalytic properties of the identified depolymerases.

The PHB degradation efficiency achieved by *Terrabacter* sp. AAH1 compares favorably with those reported for other actinobacterial PHB degraders. For instance, two *Streptomyces* species isolated from rice field soil achieved PHB degradation efficiencies of 74.5 and 68.5%, respectively, under comparable laboratory conditions (Chhetri et al., 2025). These comparisons highlight the high-efficiency degradation capacity of *Terrabacter* sp. AAH1 among actinobacteria and underscore its potential as a high-performance biocatalyst for PHB biodegradation.

## Data Availability

The datasets presented in this study can be found in online repositories. The names of the repository/repositories and accession number(s) can be found in the article/Supplementary material.

## Footnotes

1
https://www.ncbi.nlm.nih.gov/

2
https://cran.r-project.org/bin/windows/base/old/4.4.2/

3
https://ggplot2.tidyverse.org/

4
www.ncbi.nlm.nih.gov/genome/annotation_prok

5
http://leb.snu.ac.kr/ubcg2

6
www.ncbi.nlm.nih.gov/assembly/

7
http://ggdc.dsmz.de/ggdc.php

8
https://www.rdkit.org

9
https://www.ebi.ac.uk/thornton-srv/databases/pdbsum/Generate.html
