# Supplementary material for: Genomic and structural insights into the high-efficiency poly(3-hydroxybutyrate) biodegradation in Terrabacter sp. AAH1
Source: Front Microbiol. 2026 Jul 30;17:1891347. doi: 10.3389/fmicb.2026.1891347 (PMC13469037; doi:10.3389/fmicb.2026.1891347)
Supplement: Supplementary file 1 [file Data_Sheet_1.docx]

Supplementary Material

## 1. Supplementary Figures

**Supplementary Figure 1.** **The geographical location and environment of sampling sites of strain *Terrabacter* sp. AAH1.** Photograph of the agricultural stream in Gyeonggi-do, Republic of Korea, where freshwater samples were collected for the isolation of PHB-degrading bacteria, including *Terrabacter* sp. AAH1.

**Supplementary Figure 2.** **Heatmap illustrating the pairwise genomic relatedness among *Terrabacter* species.** The lower-left triangular matrix (red gradient) represents ANI values, whereas the upper-right triangular matrix (purple gradient) displays dDDH values.

**Supplementary Figure 3. Extracellular enzymatic activities of *Terrabacter* sp. AAH1.** Hydrolysis zones formed by strain AAH1 on (A) R2A agar supplemented with 2.8% (w/v) skim milk for protease activity, (B) R2A agar supplemented with 1.0% (w/v) soluble starch stained with 0.1 N iodine solution for amylase activity, and (C) R2A agar supplemented with 1.0% (w/v) carboxymethyl cellulose stained with Congo red for cellulase activity. Clear zones surrounding the colony indicate positive enzymatic activity. Plates were incubated at 30°C for 4 days.

**Supplementary Figure 4.** **TEM image of *Terrabacter* sp. AAH1 after incubation at 30℃ for 4 days.** Images were acquired using a JEM-1010 microscope (JEOL Ltd., Tokyo, Japan).

**Supplementary Figure 5. Factors influencing PHB degradation by *Terrabacter* sp. AAH1 on R2A agar supplemented with 0.1% (w/v) PHB emulsion. (A)** Effects of different culture media, including R2A, MA, TSA, LB, and NA. **(B)** Effects of temperature. **(C)** Effects of NaCl concentration. **(D)** Effects of pH. **(E)** Effects of supplementing R2A agar plates containing a PHB emulsion with 1% (w/v) carbon sources (fructose, glucose, glycerol, sucrose, or lactose) on the radius of the clear zone. Data are presented as mean ± SD of three independent replicates (n = 3).

**Supplementary Figure 6.** **Circos circular genomic map of *Terrabacter* sp. AAH1.** The genomic is visualized as concentric tracks arranged from the outermost to the innermost as follows: **(1)** genomic positions of 27 key proteins involved in PHB degradation and carbon metabolic pathways; **(2)** functional annotation of CDSs based on COG categories; **(3)** distribution of COG categories I (lipid transport and metabolism) and E (amino acid transport and metabolism), which are strongly associated with the metabolic potential of the strain; **(4)** locations of predicted rRNA and tRNA genes annotated by PGAP; note that accurate rRNA copy number determination is limited by short-read assembly; **(5)** G+C content; and **(6)** GC skew. The color-coded legend for COG classifications is shown on the right. Track 3 highlights categories I and E, containing a significant portion of genes involved in substrate assimilation.

**Supplementary Figure 6.** **Predicted substrate-binding pockets and docking grid boxes used for molecular docking.** Putative substrate-binding pockets were predicted using Fpocket for the ColabFold-generated structures of *Te*_EPD, *Te*_YbfF, *Te*_AES1, and *Te*_AES2. Protein structures are shown in cartoon representation, with **(A)** *Te*_EPD colored salmon, **(B)** *Te*_YbfF green, **(C)** *Te*_AES1 forest green, and **(D)** *Te*_AES2 lime. The selected substrate-binding pockets are shown as white surface representations, while the corresponding docking grid boxes used for AutoDock Vina calculations are shown in red. Catalytic residues are displayed as stick models. Docking grids were defined to encompass the selected binding pocket and catalytic residues for each protein.

**Supplementary Figure 7.** **Structural and sequence analyses of putative PHB depolymerases in *Terrabacter* sp. AAH1.** **(A, E, I, M)** MSAs of the modeled proteins *Te*_EPD, *Te*_YbfF, *Te*_AES1, and *Te*_AES2 with their respective homologous esterases identified using BLAST analysis. Red triangles indicate conserved substrate-binding residues, while red boxes highlight residues comprising the S–D–H catalytic triad. **(B, F, J, N)** ColabFold-predicted three-dimensional structures of *Te*_EPD, *Te*_YbfF, *Te*_AES1, and *Te*_AES2, respectively. Dashed red circles indicate N-terminal sequence extensions present in *Te*_EPD and *Te*_AES2. **(C, G, K, O)** Structural superposition of each modeled protein with its corresponding homologous esterases, illustrating conservation of the overall fold. In the structural superpositions, *Te*_EPD is shown in salmon, *AS*_FeaE in yellow, and *AL*_AXE in slate; *Te*_YbfF in green, EprEST in magenta, and *E. coli* YbfF in cyan; *Te*_AES1 in forest green, *Pv*_Est8 in sky blue, and *PC*_EST in warm pink; and *Te*_AES2 in lime, *E. coli* AES in deep teal, and *PC*_EST in hot pink. **(D, H, L, P)** Superposition of substrate-binding sites and S–D–H catalytic triads between the putative PHB depolymerases and their homologous esterases.

**Supplementary Table 1.** **Genes identified in the *Terrabacter* sp. AAH1 genome involved in PHB degradation and metabolic assimilation.**

| Gene name | Locus tag | Annotated product | Enzyme commission |
| --- | --- | --- | --- |
| **PHB depolymerization and oligomer hydrolysis** | | | |
| *Te_*EPD | BLOJFJFB_02657 | Putative extracellular esterase  (PHB depolymerase) | – |
| *Te_*YbfF | BLOJFJFB_03442 | Putative esterase | – |
| *Te_*AES1 | BLOJFJFB_01892 | Putative acetyl esterase | – |
| *Te_*AES2 | BLOJFJFB_03798 | Putative acetyl esterase | – |
| **Oxidation and Activation** | | | |
| *bdhA* | BLOJFJFB_03266 | D-beta-hydroxybutyrate  dehydrogenase | 1.1.1.30 |
| *scoA* | BLOJFJFB_03343 | Putative succinyl-CoA:3-ketoacid coenzyme A transferase subunit A | 2.8.3.5 |
| *scoB* | BLOJFJFB_03342 | Putative succinyl-CoA:3-ketoacid  coenzyme A transferase subunit B | 2.8.3.5 |
| *acsA_1* | BLOJFJFB_02408 | Acetyl-coenzyme A synthetase | 6.2.1.17 |
| *acsA_2* | BLOJFJFB_03715 | Acetyl-coenzyme A synthetase | 6.2.1.1 |
| *acsA_3* | BLOJFJFB_04076 | Acetyl-coenzyme A synthetase | 6.2.1.1 |
| **β-Oxidation-like pathway** | | | |
| *fadA* | BLOJFJFB_03462 | 3-ketoacyl-CoA thiolase | 2.3.1.9 |
| *fadI* | BLOJFJFB_03199 | Putative 3-ketoacyl-CoA thiolase | – |
| *fadB* | BLOJFJFB_01400 | 3-hydroxyacyl-CoA dehydrogenase | 2.3.1.9 |
| *fadJ_1* | BLOJFJFB_00149 | Fatty acid oxidation complex  subunit alpha | 1.1.1.157 |
| *fadJ_2* | BLOJFJFB_03240 | Fatty acid oxidation complex  subunit alpha | 1.1.1.35, 4.2.1.17,  5.1.2.3 |
| *echA8* | BLOJFJFB_00124 | Putative enoyl-CoA  hydratase echA8 | 4.2.1.17 |
| **TCA cycle and energy metabolism** | | | |
| *gltA2* | BLOJFJFB_00348 | Citrate synthase 1 | 2.3.3.1 |
| *acnA* | BLOJFJFB_02226 | Aconitate hydratase A | 4.2.1.3 |
| *icd* | BLOJFJFB_03111 | Isocitrate dehydrogenase  NADP | 1.1.1.42 |
| *kgd* | BLOJFJFB_00199 | Multifunctional 2-oxoglutarate  metabolism enzyme | 1.2.4.2, 4.1.1.71 |
| *sucC* | BLOJFJFB_03866 | Succinate–CoA ligase [ADP-  forming] subunit beta | 6.2.1.5 |
| *sucD* | BLOJFJFB_03865 | Succinate–CoA ligase [ADP-  forming] subunit alpha | 6.2.1.5 |
| *sdhA* | BLOJFJFB_03593 | Fumarate reductase flavoprotein  C-term | 1.3.5.1, 1.3.5.4, 1.4.3.16 |
| *sdhB* | BLOJFJFB_03589 | Fumarate reductase iron sulfur  subunit | 1.3.5.1, 1.3.5.4 |
| *fumB* | BLOJFJFB_00391 | Fumarate hydratase class I | 4.2.1.2, 4.2.1.32 |
| *fumC* | BLOJFJFB_02854 | Fumarate hydratase class II | 4.2.1.2 |
| *mdh_2* | BLOJFJFB_03521 | Malate dehydrogenase | 1.1.1.37 |

Functional assignments are based on sequence homology to experimentally characterized orthologs unless otherwise noted. Entries annotated as 'Putative' reflect functional predictions derived primarily from bioinformatic analyses, including KEGG/eggNOG annotation, COG classification, InterProScan domain assignment, and (where applicable) structural modeling, and have not been directly validated in *Terrabacter* sp. AAH1. Genes encoding core central metabolic enzymes (TCA cycle, β-oxidation) for which extensive experimental evidence exists in closely related taxa are retained without the 'Putative' qualifier. EC numbers were assigned based on sequence homology and KEGG orthology.

**Supplementary Table 2. Genes identified in the *Terrabacter* sp. AAH1 genome involved in PHB degradation and metabolic assimilation.**

| Gene name | Completeness (%) | Contamination (%) |
| --- | --- | --- |
| Te*rracoccus luteus* DSM 44267^T^ | 99.87 | 0.26 |
| *Terrabacter* *tumescens* JCM 1365^T^ | 99.97 | 0.14 |
| *Terrabacter* *terrigena* CCUG 57508^T^ | 100 | 0 |
| *Terrabacter* *terrae* JCM 14283^T^ | 99.74 | 0.94 |
| *Terrabacter* sp. Ter38 | 100 | 0 |
| *Terrabacter* sp. Soil811 | 100 | 0.34 |
| *Terrabacter* sp. Soil810 | 100 | 0.44 |
| *Terrabacter* sp. Root85 | 100 | 0.14 |
| *Terrabacter* sp. Root181 | 100 | 0.23 |
| *Terrabacter* sp. RAF57 | 99.99 | 0.51 |
| *Terrabacter* sp. NPDC000476 | 100 | 0.27 |
| *Terrabacter* sp. MAHUQ-38 | 100 | 0.26 |
| *Terrabacter* sp. LjRoot27 | 99.99 | 0.16 |
| *Terrabacter* sp. GCM10028922 | 100 | 0.32 |
| *Terrabacter* sp. GCM10012305 | 100 | 0.29 |
| *Terrabacter* sp. C0L 2 | 99.99 | 0.21 |
| *Terrabacter* sp. BE26 | 99.99 | 0 |
| *Terrabacter* sp. AAH1 | 99.96 | 0 |
| *Terrabacter* sp. 2YAF2 | 100 | 0.06 |
| *Terrabacter* sp. 2TAF16 | 99.99 | 0.06 |
| *Terrabacter* sp. 2RAF25 | 99.97 | 0.08 |
| *Terrabacter* *lapilli* JCM 15628^T^ | 99.82 | 0.06 |
| *Terrabacter ginsenosidimutans* JCM 17125^T^ | 100 | 0.12 |
| *Terrabacter* *carboxydivorans* JCM 16259^T^ | 100 | 0.29 |
| *Terrabacter* *aerolatus* NBRC 106305^T^ | 100 | 0.46 |
| *Terrabacter aeriphilus* JCM 17687^T^ | 100 | 0.13 |
| *Pedococcus ginsenosidimutans* JCM 18961^T^ | 100 | 1.44 |
| *Pedococcus dokdonensis* DSM 22329^T^ | 99.98 | 0.8 |
| *Pedococcus cremeus* CGMCC 1.6963^T^ | 99.91 | 0 |
| *Pedococcus badiiscoriae* DSM 23987^T^ | 98.82 | 0 |
| *Pedococcus aerophilus* JCM 16378^T^ | 100 | 0 |
| *Intrasporangium zincisolvens* YIM S08009^T^ | 100 | 0.2 |
| *Intrasporangium oryzae* NRRL B-24470 | 100 | 0.02 |
| *Intrasporangium flavum* MUSC 78^T^ | 81.31 | 0.07 |
| *Intrasporangium chromatireducens* Q5-1^T^ | 99.99 | 0.3 |
| *Intrasporangium calvum* DSM 43043^T^ | 100 | 0.01 |
| *Humibacillus xanthopallidus* DSM 21776^T^ | 100 | 0.11 |

**Supplementary Table 3**. **Grid parameters used for molecular docking of PHB trimer with the four esterase candidates.** Docking grids were defined based on substrate-binding pockets predicted by Fpocket. Grid centers (x, y, z) and dimensions (size_x, size_y, and size_z, Å) were individually optimized for each receptor to encompass the predicted binding pocket and catalytic residues.

| Protein | Grid center (x, y, z) | Grid size (x, y, z, Å) |
| --- | --- | --- |
| *Te_*EPD | 6.300, -2.935, -11.292 | 15, 16, 16 |
| *Te_*YbfF | 1.802, 5.581, -7.512 | 20, 24, 18 |
| *Te_*AES1 | 0.278, -12.455, 0.756 | 24, 21, 20 |
| *Te_*AES2 | 1.028, -9.246, -2.915 | 20, 18, 24 |

**Supplementary Table 4**. **Structural similarity between modeled esterases and homologous proteins determined by structural superposition.** Root-mean-square deviation (RMSD) values were calculated from pairwise structural alignments between each ColabFold-predicted esterase candidate and its corresponding homologous esterases with experimentally determined structures. Lower RMSD values indicate greater structural similarity.

| Query protein | Homolog | RMSD (Å) |
| --- | --- | --- |
| *Te*_EPD | *AS*_FeaE | 0.972 |
| *Te*_EPD | *AL*_AXE | 1.082 |
| *Te*_YbfF | *E. coli_*YbfF | 1.187 |
| *Te*_YbfF | EprEST | 1.146 |
| *Te*_AES1 | *PC*_EST | 0.644 |
| *Te*_AES1 | *Pv*_Est8 | 0.699 |
| *Te*_AES2 | *PC*_EST | 1.516 |
| *Te*_AES2 | *E. coli_*AES | 1.643 |

**Supplementary Table 5**. **Binding affinities of the PHB trimer to putative PHB depolymerases.**

| Protein | Binding affinity (kcal/mol) |
| --- | --- |
| Control  (PDB ID: 2D81) | -5.6 |
| *Te_*EPD | -4.9 |
| *Te_*YbfF | -6.8 |
| *Te_*AES1 | -6.1 |
| *Te_*AES2 | -5.5 |
